# A nanozyme-functionalized bilayer hydrogel scaffold for modulating the inflammatory microenvironment to promote osteochondral regeneration

**DOI:** 10.1186/s12951-024-02723-x

**Published:** 2024-07-28

**Authors:** Chuan Hu, Ruipeng Huang, Jiechao Xia, Xianjing Hu, Dingqi Xie, Yang jin, Weiming Qi, Chengliang Zhao, Zhijun Hu

**Affiliations:** 1 Department of Orthopaedic Surgery, Key Laboratory of Musculoskeletal System Degeneration and Regeneration Translational Research of Zhejiang Province, Sir Run Run Shaw Hospital, Medical College of Zhejiang University, Hangzhou, 310016 China; 2 Department of Orthopaedic Surgery, the Affiliated Hospital of Qingdao University, Qingdao, 266000 China; 3 The Second Affiliated Hospital of Wenzhou Medical University, Wenzhu, 325000 China; 4 Zhejiang Center for Medical Device Evaluation, Zhejiang Medical Products Administration, Hangzhou, 310009 China

**Keywords:** Inflammatory regulation, Osteochondral regeneration, Nanozyme, Hydrogel

## Abstract

**Background:**

The incidence of osteochondral defects caused by trauma, arthritis or tumours is increasing annually, but progress has not been made in terms of treatment methods. Due to the heterogeneous structure and biological characteristics of cartilage and subchondral bone, the integration of osteochondral repair is still a challenge.

**Results:**

In the present study, a novel bilayer hydrogel scaffold was designed based on anatomical characteristics to imitate superficial cartilage and subchondral bone. The scaffold showed favourable biocompatibility, and the addition of an antioxidant nanozyme (LiMn_2_O_4_) promoted reactive oxygen species (ROS) scavenging by upregulating antioxidant proteins. The cartilage layer effectively protects against chondrocyte degradation in the inflammatory microenvironment. Subchondral bionic hydrogel scaffolds promote osteogenic differentiation of rat bone marrow mesenchymal stem cells (BMSCs) by regulating the AMPK pathway in vitro. Finally, an in vivo rat preclinical osteochondral defect model confirmed that the bilayer hydrogel scaffold efficiently promoted cartilage and subchondral bone regeneration.

**Conclusions:**

In general, our biomimetic hydrogel scaffold with the ability to regulate the inflammatory microenvironment can effectively repair osteochondral defects. This strategy provides a promising method for regenerating tissues with heterogeneous structures and biological characteristics.

**Graphical abstract:**

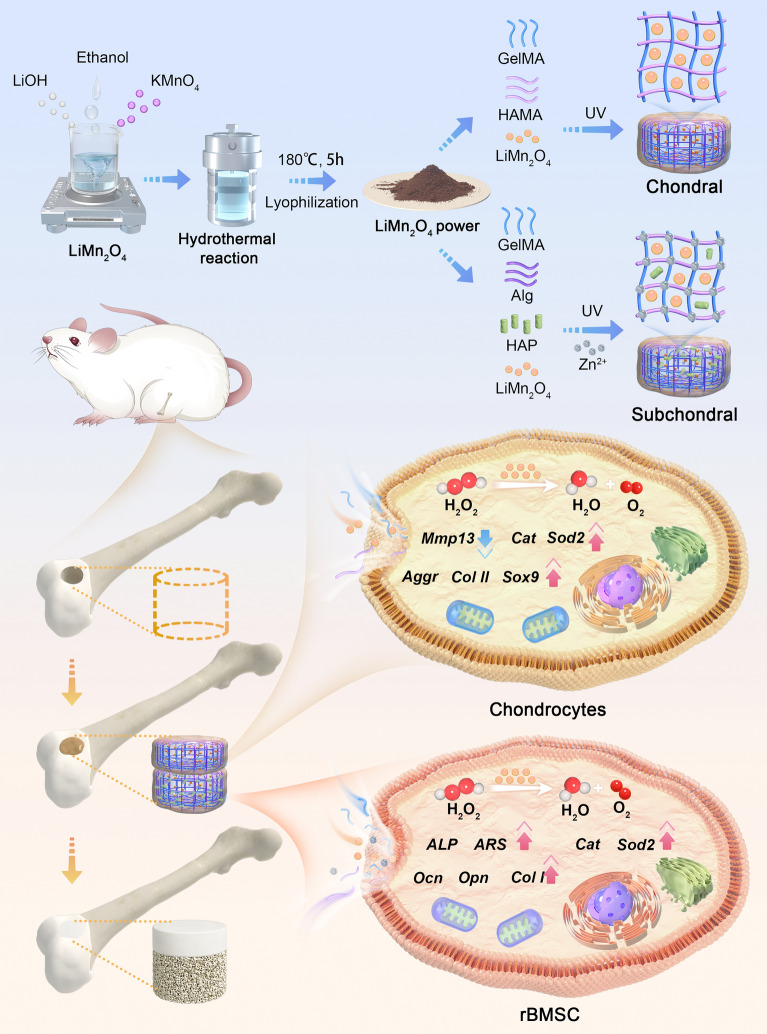

**Supplementary Information:**

The online version contains supplementary material available at 10.1186/s12951-024-02723-x.

## Background

Osteochondral defects are common and challenging diseases in clinical practice, especially in osteoarthritis (OA) patients. According to recent epidemiological reports, globally, 595 million (95% uncertainty interval 535–656) people had osteoarthritis in 2020, equal to 7.6% (95% UI 6.8–8.4) of the global population, and an increase of 132.2% (130.3–134.1) in total cases since 1990 [1]. In these patients, the continuous development of the disease leads to continuous damage to the cartilage and subchondral bone, which in turn exacerbates symptoms such as joint pain, dysfunction, and deformities, seriously affecting daily life. Even worse, the difficulty of self-repair makes external intervention necessary to treat osteochondral defects.

Articular cartilage and subchondral bone are two kinds of tissues. Articular cartilage mainly contains type II collagen and hyaline chondrocytes, while subchondral bone mainly contains type I collagen and bone tissue [2]. In addition, there are significant differences between them in terms of spatial structure [2]. Therefore, traditional single repair hydrogel scaffolds or 3D-printed scaffolds have difficulty meeting the needs of osteochondral defect repair [3–6]. In recent years, intergradient materials, including double-layer, three-layer, four-layer and even multilayer gradient scaffolds, have been gradually developed for the repair of osteochondral defects [7–13]. However, none of the scaffolds developed at present have been further promoted for clinical application, possibly due to insufficient osteochondral repair ability, difficulty in guaranteeing biosafety or immature preparation technology. In addition, the microenvironment of osteochondral defects is often in a state of oxidative stress, which is harmful to tissue repair. Therefore, pure osteogenic or chondrogenic drugs do not achieve good results. Therefore, developing a kind of multifunctional gradient scaffold that can be produced stably in batches and has superior biosafety and osteochondral repair ability is highly clinically significant.

Nanozymes are multifunctional nanomaterials discovered in recent years that can carry out multiple enzymatic functions, such as catalase (CAT), peroxidase (POD) and superoxide dismutase (SOD) activities. Utilizing these activities of nanozymes is therefore a promising avenue for treating diseases in the biomedical field and has already shown potential value in the treatment of diseases such as cancer, atherosclerosis, cancer, osteoarthritis, gout, fungal infections, atherosclerosis, wound healing and rheumatoid arthritis [14–21]. Nanozymes with great application potential are considered effective drugs. Although there are different types of nanozymes, including metal oxides, single-atom nanozymes, bimetallic nanozymes and trimetallic nanozymes, their most important roles can be divided into two main categories: the elimination of reactive oxygen species (ROS) and the production of ROS [22–29]. Thus, designing suitable nanozymes for different diseases has important clinical translational potential. However, the batch effect, biocompatibility and catalytic activity of nanozymes have been controversial issues.

**Scheme 1:**
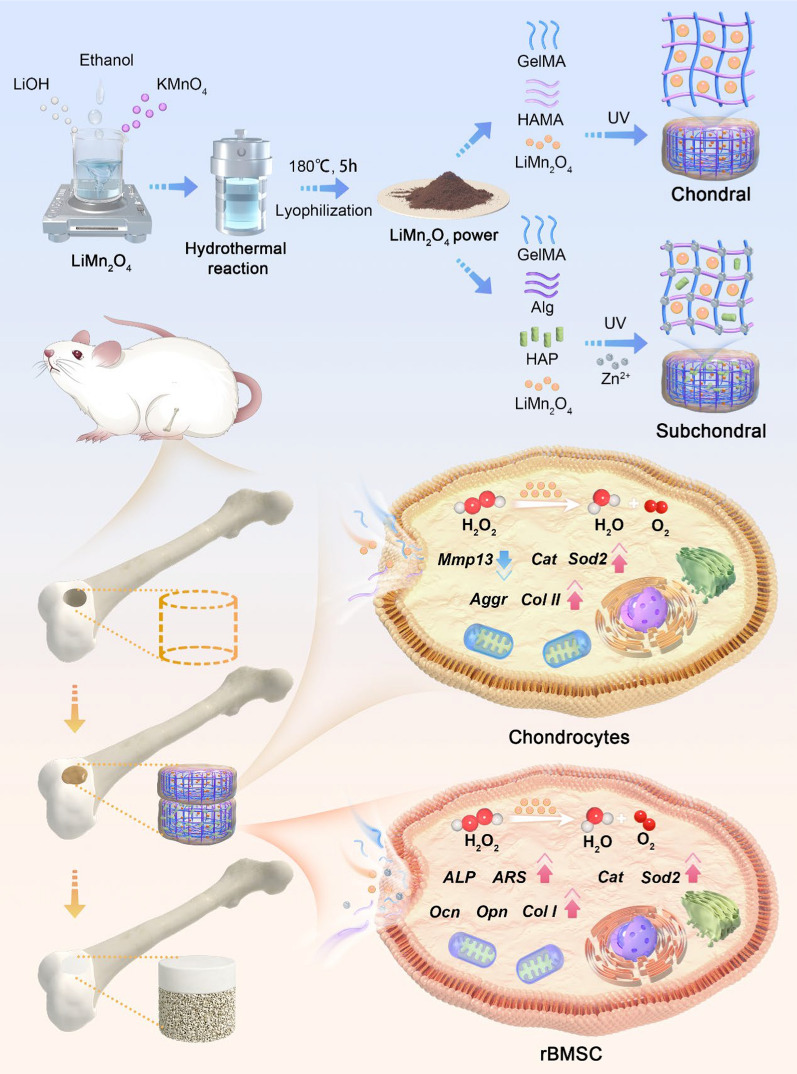
Illustration diagrams of the ability of LiMn_2_O_4_-functionalized bilayered hydrogel scaffolds to repair osteochondral defects

Recently, a valence-engineered self-cascading antioxidant nanozyme (LiMn_2_O_4_) was constructed by Wang et al. and was used for the treatment of inflammatory bowel disease (IBD) [30]. Mechanistically, LiMn_2_O_4_ has a powerful ROS scavenging ability and considerable biocompatibility, so it is worth studying whether LiMn_2_O_4_ can be used to treat osteochondral defects. Here, we report a multifunctional bilayered scaffold for osteochondral repair. The cartilage layer included the hydrogel structure of HAMA and GelMA, and LiMn_2_O_4_ was added to perform as an antioxidant nanozyme. The subchondral bone layer is composed of sodium alginate (Alg) and GelMA hydrogels, and nanohydroxyapatite (Hap) and LiMn_2_O_4_ were added to promote ossification and antioxidation, respectively. We studied the biocompatibility, anti-inflammatory performance, and chondrogenic and osteogenic properties of the hydrogel scaffold in vitro and validated the regeneration of cartilage and subchondral bone in an OC defect rat model (Scheme 1).

## Methods

### Materials

LiOH, ZnSO4, hydroxyapatite and methacrylic anhydride were purchased from Aladdin Chemical Reagent Co., Ltd. Alg, KMnO_4_ and ethanol were purchased from Guoyao Group Chemical Reagent Co., Ltd. Gelatin was purchased from Sigma‒Aldrich. DMEM/F12 and α-MEM were obtained from Zhejiang Cienry Biotechnology Co., Ltd. Fetal bovine serum (FBS) was obtained from CellMax (Peking, China). Trypsin–EDTA, Mounting Medium, and antifading agent (with DAPI) were obtained from Beijing Solarbio Science & Technology Co., Ltd.

### Synthesis and characterization of the LiMn_2_O_4_ nanozyme

The methods for preparing LiMn_2_O_4_ referred to the published literature with minor modifications [30]. Briefly, a total of 119.75 mg of anhydrous LiOH was added to 50 mL of deionized water and stirred for 10 min. Then, 0.6405 g KMnO_4_ was added to the above solution, and the mixture was stirred for 10 min. Finally, 630 μL of absolute ethanol was added to the mixed solution, the mixture was transferred to a Teflon-lined stainless-steel vessel, and the autoclave was sealed in air and maintained at 180 °C for 5 h. The obtained solution was centrifugally washed three times, and the final product was obtained by drying. Transmission electron microscopy (TEM) (HT-7700) was used to observe the morphology of the nanoparticles. The phase of the synthesized nanoparticles was analysed using X-ray diffraction (XRD) (Rigaku SmartLab SE, Japan). The valence of manganese in LiMn_2_O_4_ was analysed using X-ray photoelectron spectroscopy (XPS) (Thermo Scientific K-Alpha, USA).

### Measurement of the multienzyme activity and radical scavenging ability of LiMn_2_O_4_

The CAT-, SOD- and GPx-like activities of LiMn_2_O_4_ were measured. CAT- and SOD-like activities were measured with a catalase assay kit (Beyotime, China) and a total superoxide dismutase assay kit with WST-8 (Beyotime, China) according to the manufacturer’s instructions. The GPx-like activity was measured with a total glutathione peroxidase detection kit (S0058, Beyuntian), and the change in absorbance of NADPH was monitored at 340 nm.

### Preparation of layered scaffold

GelMA was prepared according to previous methods, and HAMA was purchased from Engineering for Life (Soochow, China). For the cartilage hydrogels, GelMA (5%) and HAMA (2%) were simultaneously dissolved in ddH_2_O supplemented with 0.1% photosensitizer and cross-linked using UV light; these materials were named GH. For GH@LM, 0.5% LiMn_2_O_4_ nanozymes were added to the above hydrogel. For the subchondral scaffold, GelMA (5%) and Alg (1%) were simultaneously dissolved in ddH_2_O supplemented with 0.1% photosensitizer. A ZnSO_4_ solution (200 mM) was used to form the first cross-linked network, and then, the primary hydrogel was cross-linked using UV light to form the second network, named GA. For GA@H and GA@HLM, 0.1% Hap or 0.1% Hap mixed with 0.5% LiMn_2_O_4_ nanozymes was added to the above hydrogel. Three types of layered scaffolds were prepared, including GH + GA, GH + GA@H and GH@LM + GA@HLM. Layered scaffolds contain 75% (lower layer) subchondral hydrogel and 25% (upper layer) cartilage hydrogel, respectively.

### Scanning electron microscopy

After lyophilization, the morphology of the hydrogel was observed by scanning electron microscopy (SEM) (SU-8010), and the elemental composition was confirmed via an X-max80.

### Swelling analysis

GH + GA, GH + GA@H and GH@LM + GA@HLM hydrogels with equal mass were prepared, weighed (W_1_) and freeze-dried. Then, the freeze-dried hydrogels were immersed in PBS solution and incubated at 37 ℃. At the set time point, the hydrogel was removed and its mass was measured (W_2_). The swelling ratio was calculated with the equation below:

$${\text{Swelling}}\,{\text{rate}}\,\left( \% \right)\, = \,\left( {{\text{W}}_{{1}} /{\text{W}}_{{2}} } \right)\, \times \,{1}00\%$$

### Degradation studies

GH + GA, GH + GA@H and GH@LM + GA@HLM hydrogels with equal mass (W_1_) were prepared and immersed in PBS. Then, hydrogels were incubated at 37 ℃. At a set point in time, hydrogels were removed and its mass was measured (W_2_). The degradation rate of hydrogel was calculated with the equation below:

$${\text{Degradation}}\,{\text{rate}}\,\left( \% \right)\, = \,\left( {\left( {{\text{W}}_{{1}} - {\text{W}}_{{2}} } \right)/{\text{W}}_{{1}} } \right)\, \times \,{1}00\%$$

### Release of LiMn_2_O_4_

A total of 1500 mg of GH@LM + GA@HLM hydrogels was immerged to 25 mL PBS at 37 ℃. At specific time points, 2 mL release medium was collected and replaced by 2 mL fresh PBS. The concentration of LiMn_2_O_4_ was calculated according to the absorbance of the release medium at 315 nm measured by UV spectrophotometer (Shimadzu, Japan).

### Mechanical testing

The mechanical properties of hydrogels were tested using a universal testing machine (Instron 5967, USA). First, GH@LM + GA@HLM hydrogel with cylindrical shape (8 mm diameter and 5 mm height). The samples were compressed at a speed of 1 mm/min until breakage.

### Cell culture and in vitro experiments

#### Isolation and culture of rat chondrocytes (RCs) and bone marrow mesenchymal stem cells (BMSCs)

RCs were harvested from 3-day-old SD rats. Briefly, the obtained cartilage tissue was washed with PBS, cut into 1 mm^3^ pieces, and incubated with 0.2% collagenase II for 12 h at 37 °C. The obtained RCs were subsequently resuspended in DMEM/F12 culture medium and filtered through a 40 μm cell filter before seeding at a density of 2 × 10^5^ cells/mL. For BMSCs, cells were isolated from the bone marrow of 4-week-old rats according to previous methods [31]. RCs were cultured in DMEM/F12 medium, and BMSCs were cultured in α-MEM. All mediums contained 10% FBS and 1% penicillin/streptomycin. Only cells in passages 2–4 were used in the present study.

#### CCK-8 assay

CCK-8 assays were also performed using RCs and BMSCs to evaluate the cytotoxicity of the nanozymes and hydrogels. Briefly, cells (5000/well) were incubated with a 96-well plate at 37 °C overnight. Then, different treatments were added to each well of the plate for another 3–5 days. At each time point, the cells were incubated in 10% CCK-8 solution for 2 h, after which the absorbance of the incubation medium was measured at 450 nm.

#### Live/dead staining

Live/dead staining was performed to evaluate the cytotoxicity of the nanozymes and hydrogels by co-culturing hydrogels with cells. RC or BMSC cells were incubated with a 24-well plate (2 × 10^4^/well) at 37 °C overnight. Then, different treatments were added to each well of the plate for another 3–5 days. At each time point, the Calcein-AM/PI Double Stain Kit (Yeasen Biotechnology, Shanghai) was added, and the images were visualized using a fluorescence microscope.

#### ROS scavenging ability

RC and BMSCs were seeded on 24-well plates at a density of 2 × 10^4^ cells/well. After 12 h, the cells cultured in the lower chamber were treated with 400 μM H_2_O_2_ for 24 h, and the NPs were added, or the hydrogels were cocultured for 5 days after they were added. After the incubation, the cells were washed in PBS three times, after which the DCFH-DA probe (Solarbio, Beijing) was added. The fluorescence in the cells was observed, and images were collected using fluorescence microscopy. For qRT‒PCR and western blotting analysis of two antioxidative biomarkers, cells were seeded on 6-well plates at a density of 8 × 10^4^ cells/well. After 12 h, the transwell inserts were added, and the hydrogels were placed in the upper part. After 5 days of coculture, cells were harvested for RNA or protein extraction for further use.

#### RNA extraction and qRT‒PCR analysis

Total RNA was extracted by AG RNAex Pro RNA reagent (Agbio, Hunan) according to the manufacturer’s instructions, and reverse transcription was performed by premix reagent for qPCR (Agbio, Hunan). Then, qRT‒PCR was carried out to evaluate the relative expression of the target mRNAs. The primers used in the present study are listed in Table S1.

#### Protein extraction and western blotting

Total protein was extracted from the RCs and BMSCs using RIPA buffer supplemented with PMSF, and the concentrations were determined via a BCA kit. Then, the proteins were separated via SDS‒PAGE and transferred onto PVDF membranes. After blocking with 5% BSA in TBST for 90 min, the membranes were incubated overnight at 4 °C with the corresponding primary antibodies, which included Gapdh (Proteintech, 60004-1-Ig), Cat (Proteintech, 66765–1-Ig) and Sod2 (Proteintech, 24127-1-AP). Then, the membranes were washed with TBST and incubated with goat anti-rabbit/mouse secondary antibodies. Immunoreactive bands were visualized using an Amersham Image 600. Band intensity was quantified using ImageJ.

#### Immunofluorescence staining of cells

The cells were seeded in chamber slides and fixed in 4% paraformaldehyde for 20 min. Then, the cells were permeabilized with 0.1% Triton X-100 and blocked with 5% BSA for 1 h at 25 °C. Primary antibodies were added, and the samples were incubated at 4 °C overnight. Then, the slides were washed three times with PBS and incubated with secondary antibodies (Fude Biological Technology Co., Ltd., China) for 60 min at room temperature. Nuclei were stained with DAPI for visualization, and digital images were taken using a digital pathology section scanner (KFBIO, China).

#### Alkaline phosphatase activity and alizarin red S staining

BMSCs were seeded onto 24-well plates at a density of 2 × 10^4^ cells/well. The cells were cultured overnight, the medium was replaced with osteogenic induction medium, and the cells were treated with different hydrogels. After 7 or 14 days, a BCIP/NBT phosphatase color development kit (C3206, Beyotime, China) was used to measure the expression of ALP in the BMSCs. In addition, for alizarin red S staining, 2% alizarin red S staining solution (C0138, Beyotime, China) was used to identify mineralization in the BMSCs.

#### RNA sequencing analysis

The cells were cultured in 6-well plates at a density of 1 × 10^5^ cells/well and treated with PBS or hydrogel for seven days. Total RNA was subsequently isolated from each well, RNA integrity was assessed via 1.5% agarose gel electrophoresis or fragmentation analysis, and RNA purity was assessed via a NanoDrop spectrophotometer. The RNA-seq libraries were constructed using a TruSeq™ RNA Sample Prep Kit, and the libraries were subjected to deep sequencing via paired-end sequencing with a NovaSeq 6000 S4 Reagent Kit according to the manufacturer’s protocol. Transcriptome sequencing was subsequently conducted by Honsunbio Biotech Co., Ltd. (Shanghai, China). The differentially expressed gene (DEG) analysis was performed using R software, and an adjusted p < 0.05 and fold change > 2 or < −2 were set as the thresholds for significantly differential expression. The gene ontology (GO) and Kyoto Encyclopedia of Genes and Genomes (KEGG) of DEGs was analysed in R and Metascape and visualized with the ggplot2 package [32].

### In vivo animal experiment

#### Surgical procedure

The animal use protocol listed below was reviewed and approved by the Institutional Animal Care and Use Committee (IACUC) of ZJCLA. (Number: ZJCLA-IACUC-20030090). The scaffold used in the animals was a cylinder (diameter: 2.0 mm, height: 2.0 mm). Eight-week-old S-D rats were used and randomly divided into control, GH + GA, GH + GA@H, and GH@LM + GA@HLM groups (n = 3/group). After anaesthesia, the femoral condyles were exposed, and an osteochondral cylindrical defect was generated using an electric surgical drill and then implanted with different hydrogels. The hydrogels used in vivo consisted of 1.5 mm subchondral bone hydrogels and 0.5 mm cartilaginous hydrogels, which contained the same concentration of drugs as in vitro. Finally, the S-D rats were allowed to move freely and were provided standard food and water. After 6 and 12 weeks of feeding, distal femurs were obtained and fixed with 4% paraformaldehyde.

#### Micro-CT evaluation

After the rat knee samples were fixed with 4% paraformaldehyde for 2 days, micro-CT (Skyscan1275) was used to scan bone information from the distal femur, and NiRcon was used for three-dimensional reconstruction. DataViewer and CTAn software were used to calculate the bone volume (BV), total volume (TV) and Tb.N. CTvox was performed to obtain three-dimensional images of the distal femur.

#### Histological examination

The dissected distal femurs were decalcified in EDTA decalcifying solution and embedded in paraffin for routine histological sectioning. Sagittal sections. 5 μm in thickness were obtained from the center of each defect and stained with H&E, Masson’s trichrome and safranin O/fast green to evaluate OC regeneration. After that, immunofluorescence staining of Col II, Col I, Aggrecan and Opn was performed to evaluate the quality of the regenerated cartilage and subchondral bone. The slices were stained with primary antibodies against Col II (Arigo, ARG20787), Aggrecan (Proteintech, 13880-1-AP), Col I (Abcam, ab270993), Opn (Proteintech, 22952-1-AP) and Sod2 (Proteintech, 24127-1-AP) overnight at 4 °C. Subsequently, the sections were further stained with secondary antibodies (Fude Biological Technology Co., Ltd., China) conjugated with fluorescent dyes. Finally, the nuclei were stained with DAPI for visualization, and immunofluorescence images were recorded with a digital pathology section scanner (KFBIO, China).

## Results and discussion

### LiMn_2_O_4_ nanozymes can effectively clear ROS and inhibit cell apoptosis

**Fig. 1:**
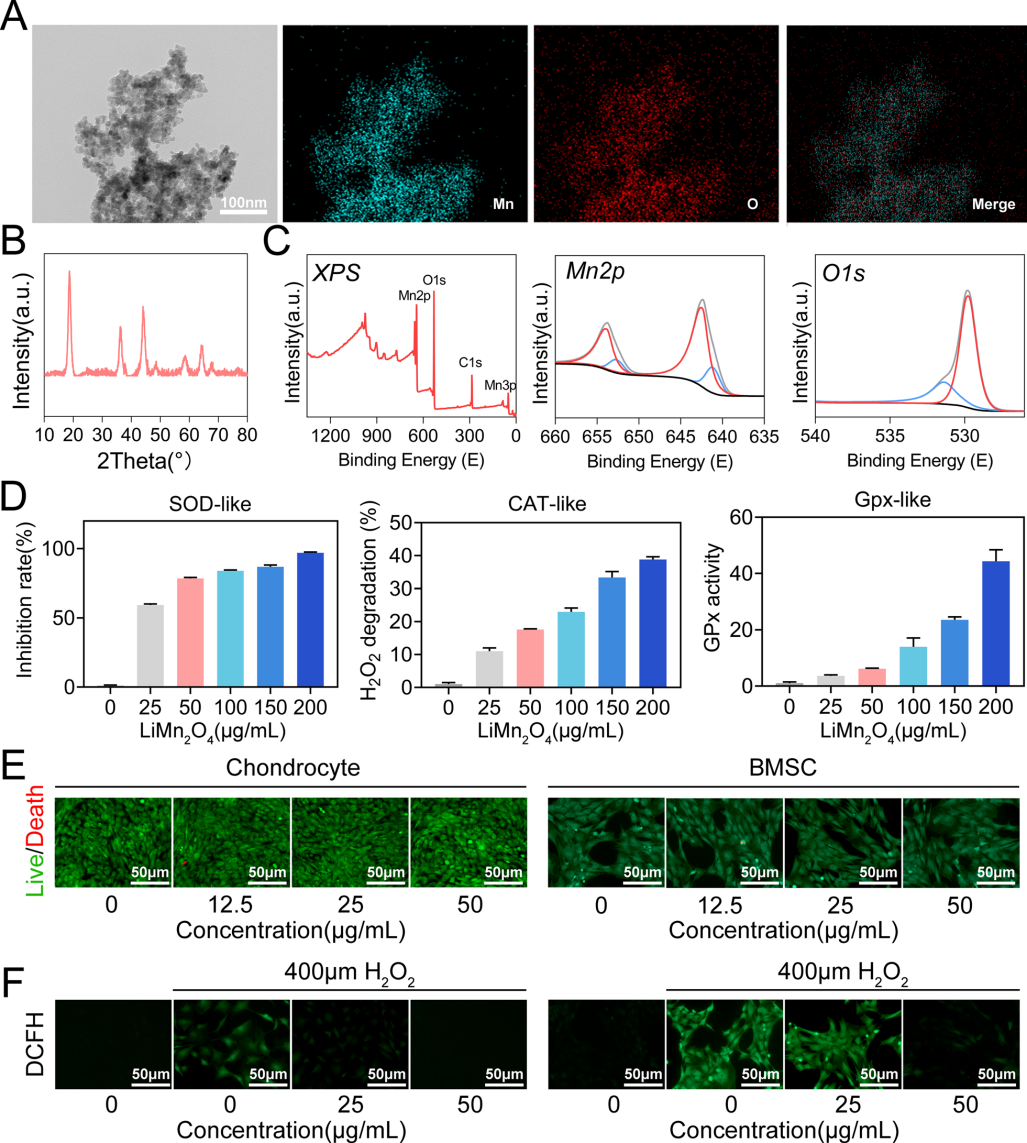
Characteristic and multiple enzyme-like activities of the LiMn_2_O_4_ nanozyme. **A** TEM and elemental mapping of LiMn_2_O_4_ nanoparticles; **B** XRD patterns of LiMn_2_O_4_ nanoparticles; **C** XPS spectrum of LiMn_2_O_4_ nanoparticles; **D** SOD-, CAT- and Gpx-like activities of LiMn_2_O_4_ nanoparticles; **E** Live/dead staining of LiMn_2_O_4_ nanoparticles in rat chondrocytes and BMSCs; **F** DCFH staining of LiMn_2_O_4_ nanoparticles in rat chondrocytes and BMSCs

LiMn_2_O_4_ nanozymes were synthesized using a hydrothermal reaction method at 180 °C (5 h), and the synthesis process of these nanoparticles is shown in Scheme 1. The TEM morphology of the obtained LiMn_2_O_4_ nanozyme is shown in Fig. 1A, and the Energy Dispersive Spectrometer (EDS) results showed that the obtained nanoparticles mainly contained Mn and O (Fig. 1A). Because Li is difficult to represent via EDS, it was not analysed here. The charge of LiMn_2_O_4_ was characterized using DLS, and the zeta potential was approximately 16.20 ± 0.66 mV (Figure S1). The XRD and XPS spectra of LiMn_2_O_4_ are shown in Fig. 1B, C. Mn and O were clearly detected. Additionally, it should be noted that the peak attributed to Li was so weak that it was covered by the Mn 3p peak [30]. Overall, these results indicated that the synthesized LiMn_2_O_4_ nanozyme is the same material as previously reported and is worthy of further biomedical application [30].

Multienzyme activity, including SOD-, CAT-, and Gpx-like activities, is important for LiMn_2_O_4_ [30]. SOD can convert ^·^O_2_^−^ to H_2_O_2_, which is subsequently converted to O_2_ and H_2_O by CAT [33]. ^·^O_2_^−^ and H_2_O_2_ are the two most influential ROS in the microenvironment of osteochondral defects and have damaging effects on tissue repair. Therefore, nanomaterials with SOD- and CAT-like activities can promote the repair of osteochondral defects by eliminating ROS [3–5]. With increasing LiMn_2_O_4_ concentration, its ability to eliminate ^**·**^O_2_^−^ (SOD-like) continued to increase, and nearly 100% clearance was reached at 200 μg/mL (Fig. 1D). CAT activity tests also showed that LiMn_2_O_4_ can effectively eliminate H_2_O_2_ (Fig. 1D). GPx catalyzes the reduction of glutathione (GSH) to reduce H_2_O_2_ and various organic peroxides, thereby eliminating the toxic effects of ROS. We further studied the function of LiMn_2_O_4_ in RCs and BMSCs. After culture medium containing different concentrations of LiMn_2_O_4_ were added to the RCs and BMSCs, live/dead staining was performed on the 5 days, and the results showed that there was no significant difference in cell viability between the groups treated with 0, 12.5, 25, or 50 µg/mL LiMn_2_O_4_, and only a small amount of cell death occurred in all the groups (Fig. 1E). DCFH fluorescence revealed that LiMn_2_O_4_ effectively scavenged ROS at a concentration of 25 μg/mL, while it almost completely scavenged ROS from RCs and BMSCs at a concentration of 50 μg/mL (Fig. 1F). Under oxidative stress, ROS accumulate continuously in periarticular cells, causing apoptosis of RCs and subchondral bone, thus impairing the function of normal joints [34, 35]. Therefore, effective scavenging of intracellular ROS can inhibit the occurrence of apoptosis and is a potential treatment for osteochondral defects. Generally, we successfully synthesized LiMn_2_O_4_ nanozymes and confirmed that they are safe and reliable potential drugs for the treatment of osteochondral defects.

### Synthesis and characterization of bilayered hydrogel

**Fig. 2:**
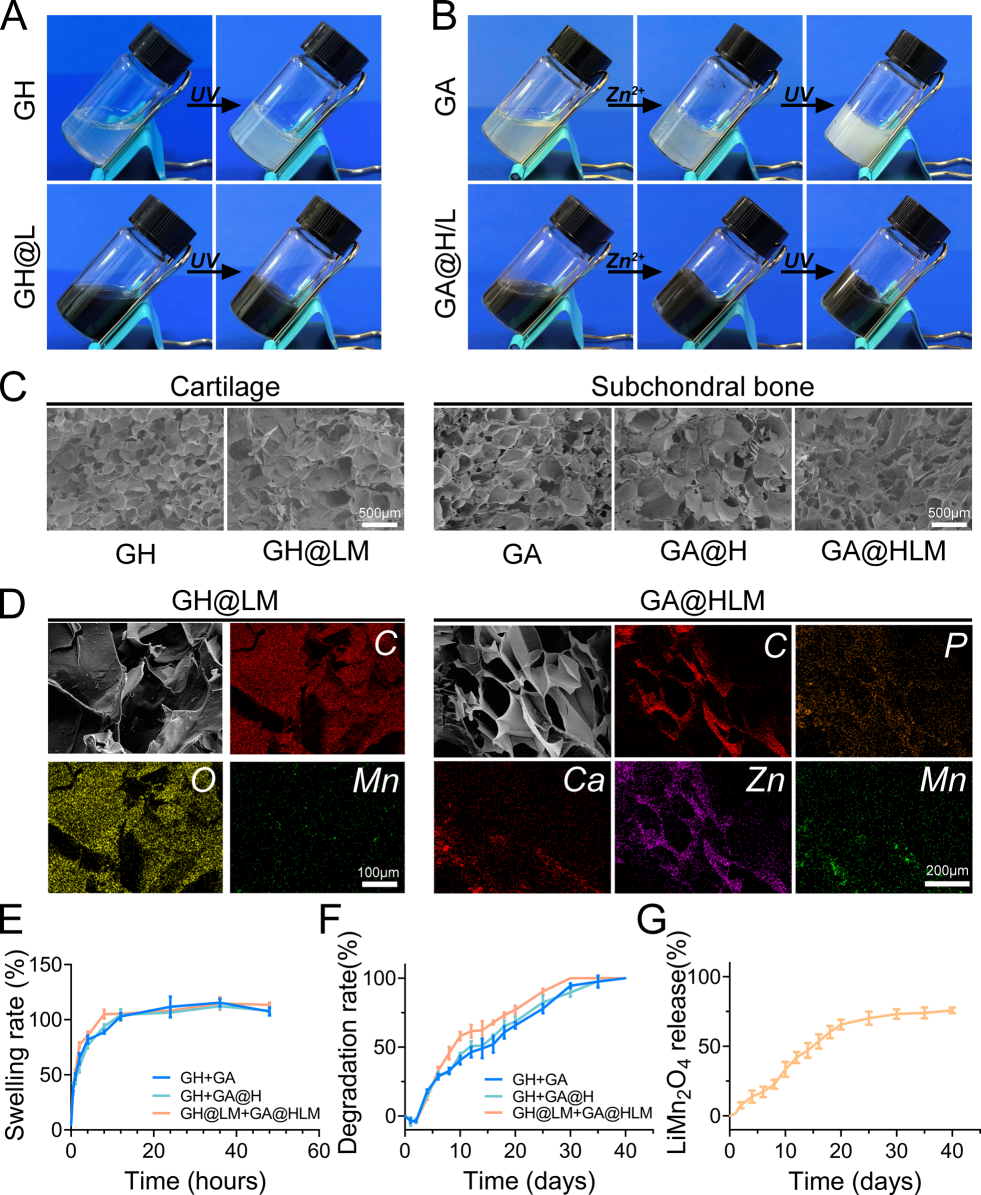
Preparation and characterization of nanozyme-functionalized bilayer hydrogel scaffolds. **A** Photos of the gel-sol transition between the GH and GH@LM hydrogels; **B** photos of the gel-sol transition between the GA and GA@HLM hydrogels; **C** SEM images of the GH, GH@LM, GA, GA@H, and GA@HLM hydrogels; **D** Elemental mappings of the GH@LM and GA@HLM hydrogels; **E** Swelling ratios of GH + GA, GH + GA@H, and GH@LM + GA@HLM hydrogels; **F** Degradation rate of GH + GA, GH + GA@H, and GH@LM + GA@HLM hydrogels; **G** LM release curve of GH@LM + GA@HLM hydrogel

The hydrogel used to treat the cartilage layer is shown in Fig. 2A. After UV irradiation, the solution changes to a gel. The hydrogel for subchondral bone formed the first cross-linked network after the addition of Zn^2+^ and the second cross-linked network after UV irradiation (Fig. 2B and S2). Obviously, the addition of LiMn_2_O_4_ makes the hydrogel rich in its dark brown colour. SEM showed that all the hydrogels displayed a porous morphology (Fig. 2C). The even distribution of manganese in the EDS images of GH@LM and GA@HLM directly confirmed the incorporation of LiMn_2_O_4_ NPs into the hydrogel network (Fig. 2D, Figure S3 and S4).

After gelation, we obtained cylindrical subchondral bone (Lower 75%) and cartilage (Upper 25%) hydrogels, respectively, and established bilayered hydrogel, including GH + GA, GH + GA@H, and GH@LM + GA@HLM (Figure S5). Then, the swelling ratio, degradation, nanoparticle release and mechanical properties of composite hydrogels were analysed. Figure 2E shows the swelling outcomes of the three hydrogels. All hydrogels rapidly absorbed liquid within the first 4 h and then slowly expanded until swelling equilibrium was reached after 12 h. Afterward, the degradation of the hydrogels was analysed. Overall, the GH@LM + GA@HLM hydrogel had almost completely broken down and degraded by day 30, and its degradation was quickly than GH + GA, GH + GA@H hydrogels (day 35) (Fig. 2F). Because LM is the nanoparticle that plays a vital role in hydrogels, we continue to study its release outcome. In GH@LM + GA@HLM hydrogel, LM was gradually released, with the release rate reaching 73.2% by Day 30 (Fig. 2G). For mechanical performance, compressive testing showed that the compressive modulus of GH@LM + GA@HLM is 73.53 kPa. These results showed that the composite hydrogel had good swelling and degradation properties, and gradually released antioxidant nanoparticles to repair osteochondral defects.

### The bilayered hydrogel showed favourable biocompatibility and intracellular ROS scavenging ability

**Fig. 3:**
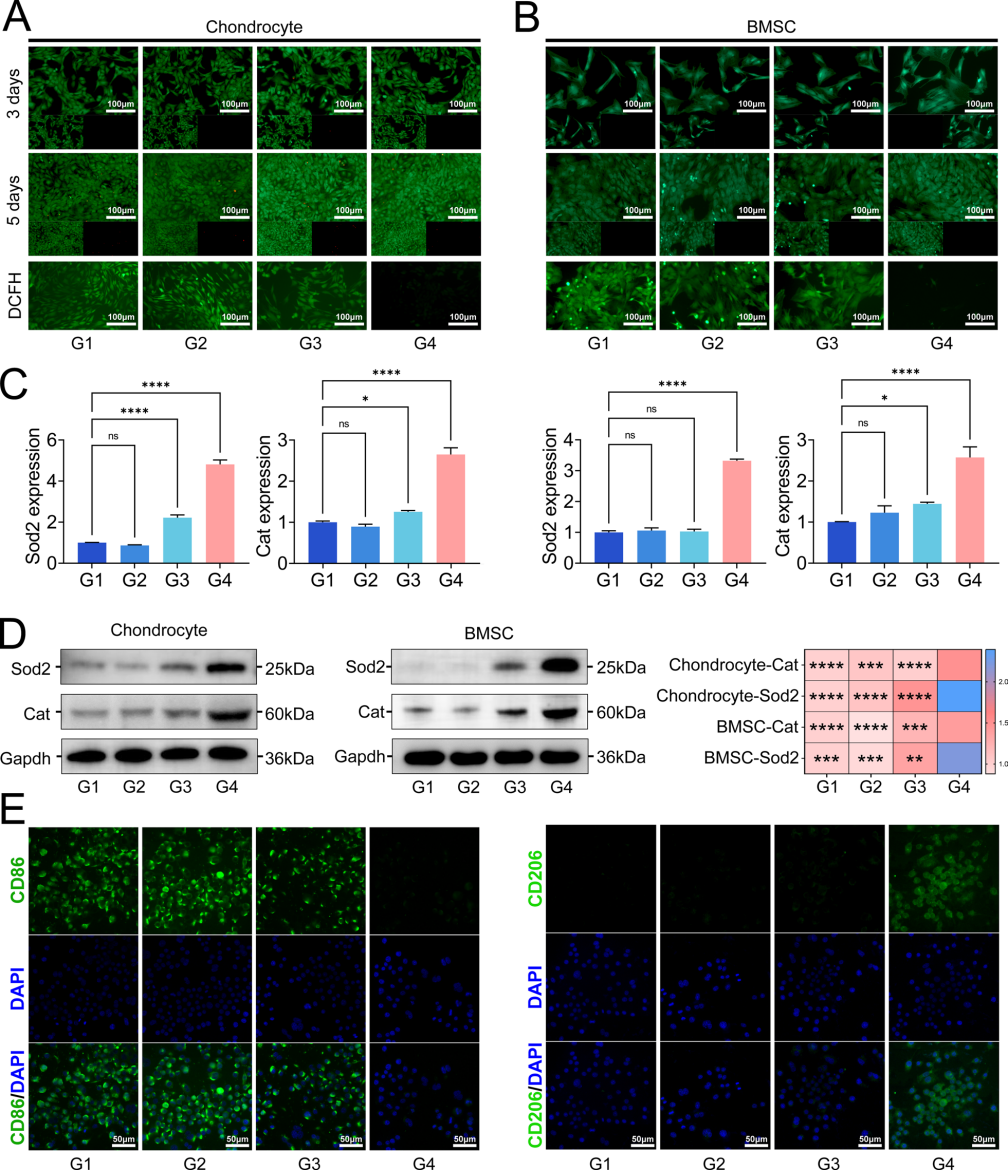
Biocompatibility and anti-inflammatory performance of the bilayer hydrogel scaffold. **A** Live/dead staining and ROS scavenging staining of bilayer hydrogel scaffolds in rat chondrocytes; **B** Live/dead staining and ROS scavenging staining of bilayer hydrogel scaffolds in rat BMSCs; **C** mRNA expression of two antioxidative genes in rat chondrocytes and BMSCs after treatment with hydrogel scaffolds; **D** Protein expression of two antioxidative genes in rat chondrocytes and BMSCs after treatment with hydrogel scaffolds; **E** Immunofluorescence of polarization markers of macrophages treated with hydrogel scaffolds. G1: PBS; G2: GH + GA; G3: GH + GA@H; G4: GH@LM + GA@HLM. The data are presented as the means ± standard deviations (SD), n = 3. (*p < 0.05; **p < 0.01; ***p < 0.001; ****p < 0.0001)

CCK8 and live/dead staining were used to evaluate the biocompatibility of the hydrogels. To better study the safety of double-layer composite hydrogels, we studied the use of double-layer hydrogels on both RCs and BMSCs. CCK8 results showed that the three hydrogels did not cause significant cellular toxicity on day 3 or day 5 (Figure S7). Although some cells died on day 5 according to the results of dead/alive staining, there was no significant difference compared with that in the control group (Fig. 3A, B). Therefore, the three hydrogels showed excellent biosafety. When stimulated with hydrogen peroxide (200 μM), both the RCs and BMSCs exhibited high fluorescence, which confirmed that hydrogen peroxide could effectively induce intracellular ROS, and the intracellular ROS could not be effectively cleared when the hydrogels were treated without the LiMn_2_O_4_ nanozyme (Fig. 3A, B). However, the fluorescence level of DCFH in the GH@LM + GA@HLM group significantly decreased, indicating that the fictionalized LiMn_2_O_4_ hydrogel effectively eliminated the intracellular ROS in the RCs and BMSCs (Fig. 3A, B). Recently, it was reported that nanozymes can exert antioxidative effects by upregulating the expression of antioxidation genes in cells; therefore, we also conducted similar research [36–39]. PCR revealed that GH@LM + GA@HLM could significantly increase the mRNA expression of two antioxidant genes, SOD2 and CAT (Fig. 3C). In addition, the protein expression levels of SOD2 and CAT were significantly increased after treatment with GH@LM + GA@HLM (Fig. 3D). Macrophages, which include mainly the M1 and M2 subtypes, play an important role in tissue repair. By scavenging intracellular ROS, the polarization of macrophages can be effectively regulated in a direction beneficial for tissue repair. Therefore, we conducted a preliminary study on the effect of our material on macrophages. Interestingly, in the presence of LiMn_2_O_4_, the hydrogel effectively promoted M2 polarization of RAW264.7 cells (Fig. 3E). The expression of the M2 macrophage marker CD206, was significantly greater in the GH@LM + GA@HLM group, while the expression of the M1 macrophage marker CD86 was significantly lower in this group (Figure S8). Overall, GH@LM + GA@HLM is a multifunctional hydrogel with excellent biosafety that can protect RCs and BMSCs from oxidative stress by increasing the expression level of intracellular antioxidant genes and regulating the immune microenvironment.

### GH@LM showed RC protection by regulating homeostasis

**Fig. 4:**
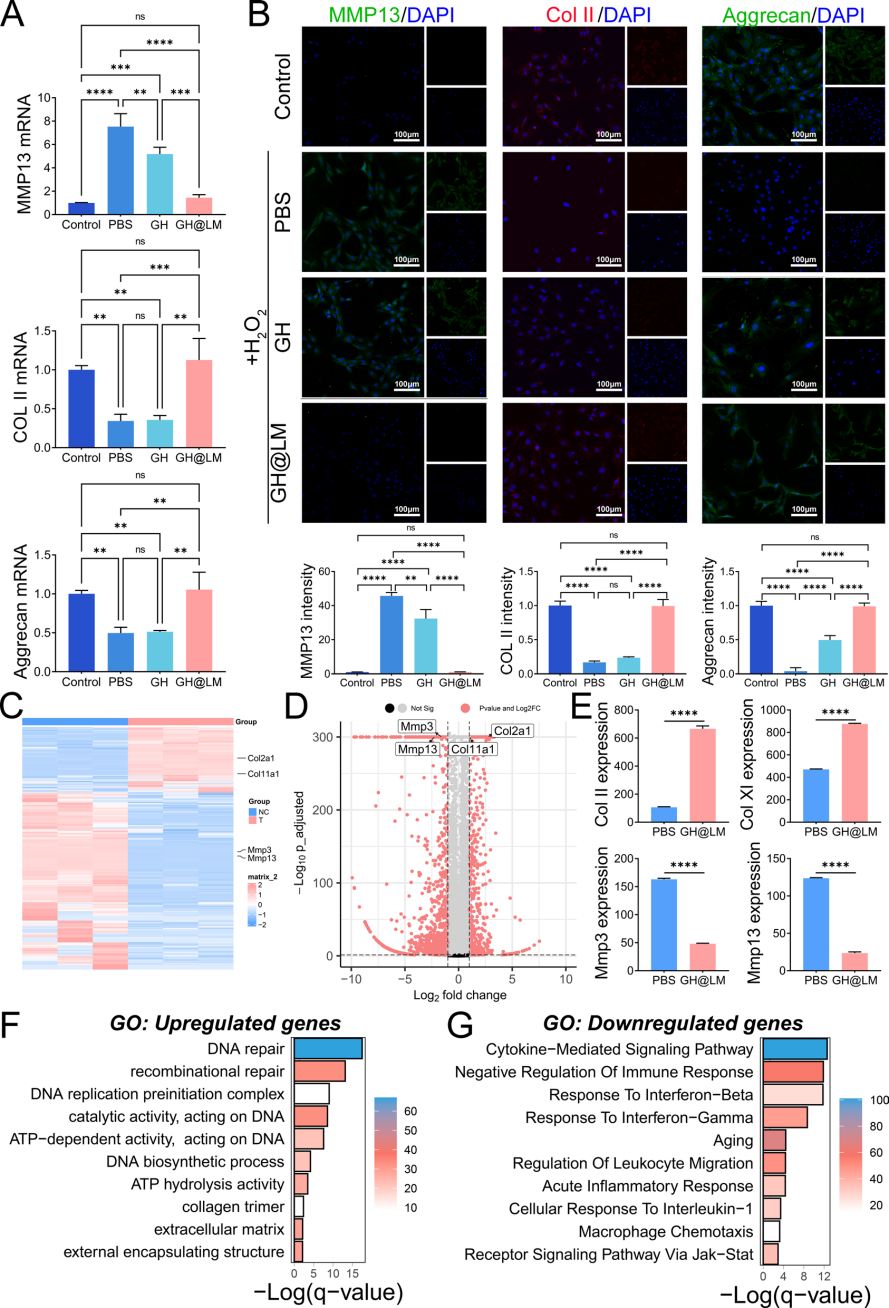
The mechanism by which cartilage layer hydrogels protect chondrocytes from ROS damage. **A** mRNA expression of Col II, Aggrecan, and Mmp13 in rat chondrocytes after treatment with different hydrogels; **B** Immunofluorescence staining and analyses of Col II, Aggrecan, and Mmp13 in rat chondrocytes after treatment with different hydrogels; **C** Heatmap showing DEGs between chondrocytes treated with PBS and the GH@LM hydrogel; **D** Volcano plot showing the differential analysis results; **E** Expression of Col II, Col XI, Mmp3 and Mmp13 in chondrocytes treated with PBS and the GH@LM hydrogel determined by RNA sequencing; **F** Representative GO items of genes upregulated in the GH@LM hydrogel-treated groups; **G** Representative GO items of genes downregulated in the GH@LM hydrogel-treated groups. The data are presented as the means ± standard deviations (SD), n = 3. (**p < 0.01; ***p < 0.001; ****p < 0.0001)

Oxidative stress is a very important pathology of cartilage injury [40–42]. We have demonstrated that the GH@LM hydrogel is effective at scavenging ROS. Therefore, we further investigated the role it plays in the process of articular cartilage protection. The mRNA expression of MMP13 in the GH@LM group was significantly lower than that in the PBS and GH groups, while the expression of Col II and Aggrecan was significantly greater in this group (Fig. 4A). Moreover, immunofluorescence exhibited a trend similar to that of PCR (Fig. 4B). Taken together, these results showed that GH@LM is a multifunctional hydrogel that inhibits chondrocyte matrix degradation and inflammation in the oxidative stress microenvironment. The mechanism of the protective effect of GH@LM was explored via transcriptome sequencing, and specimen correlation testing showed that there was a high correlation between three replicate samples in each group (Figure S9). By comparing the expression between the GH@LM-treated group and the PBS-treated group, 2904 genes were confirmed to be dysregulated in the GH@LM group. A total of 2122 and 782 genes were downregulated and upregulated, respectively, in the GH@LM group (Fig. 4C, D). Like the PCR and immunofluorescence results, the RNA sequencing results showed that inflammatory genes, such as Mmp3 and Mmp13, were significantly downregulated after GH@LM treatment, while cartilage matrix-related genes, such as Col2a1 and Col11a1, were significantly upregulated (Fig. 4C, D, and E). The protein–protein interaction (PPI) results are shown in Figure S10, and many inflammatory genes, such as Mmp3, Ccl2, Il-10, and Ccl5, were confirmed to be hub genes. The enrichment analyses of the DEGs were persuasive. The genes upregulated in the GH@LM treatment group were associated mainly with collagen, the extracellular matrix, and energy metabolism, which is meaningful for the survival of patients in the RCs (Fig. 4F). The genes downregulated in the GH@LM treatment group were significantly associated with interferon, interleukin-1, aging, and cytokines (Fig. 4G). KEGG pathway analysis also revealed the potential mechanism of action of GH@LM on cartilage from other perspectives (Figure S11 and S12). The osteoclast-chondrocyte interaction is a newly confirmed cellular communication pathway that can regulate chondrocyte apoptosis by blocking osteoclast-related receptor (OSCAR) to prevent articular cartilage destruction [43]. In the present research, the KEGG pathway analysis results indicated that the genes downregulated in the GH@LM group were significantly associated with osteoclast differentiation, which may be a direction worthy of further investigation into the mechanism of our hydrogel scaffold in the future. Overall, these findings confirmed that GH@LM can effectively protect chondrocytes in an oxidative stress microenvironment to promote the repair of cartilage defects.

### GA@HLM strongly promoted osteogenic activity in an oxidative stress microenvironment by regulating the AMPK pathway

**Fig. 5:**
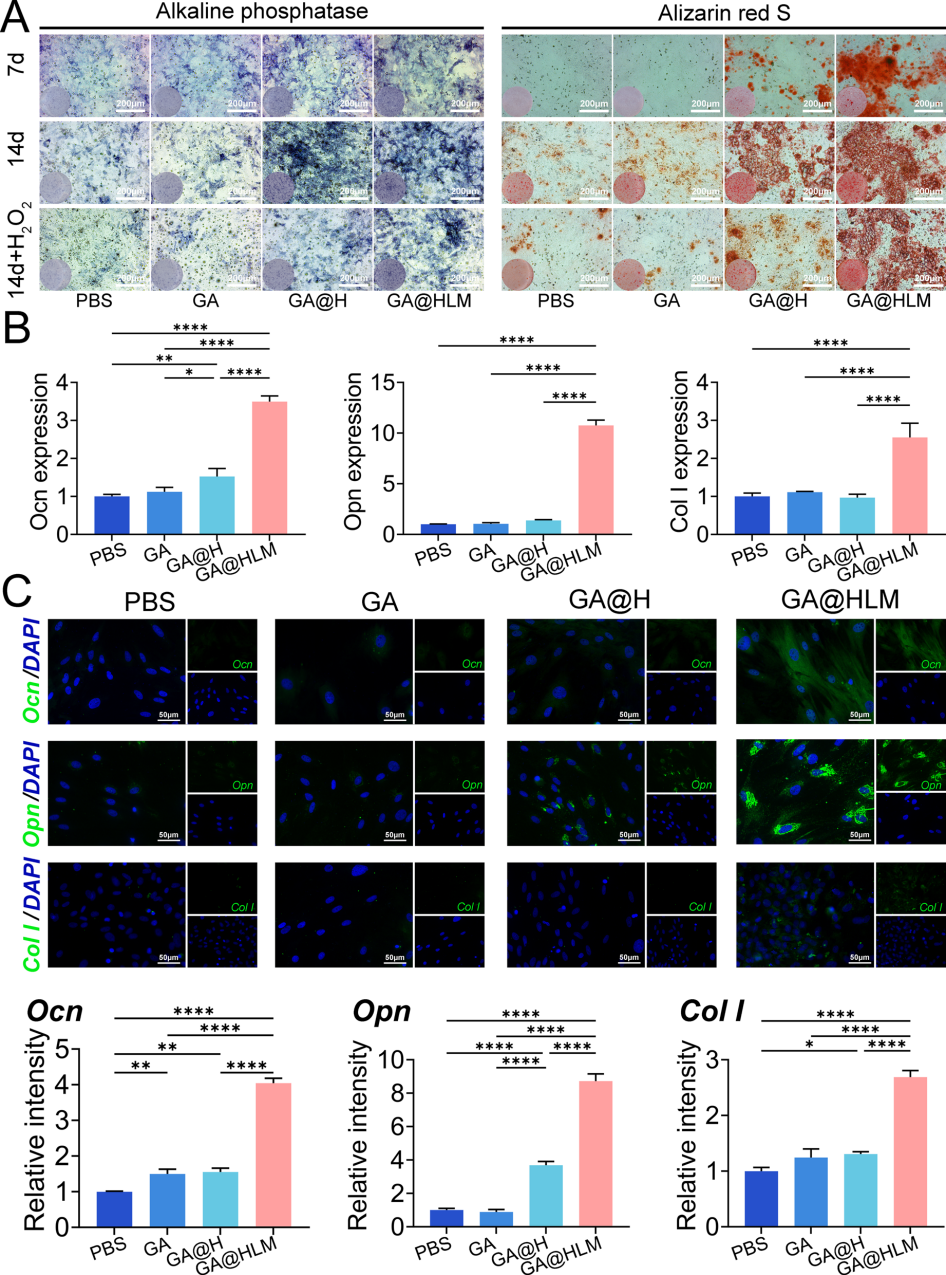
The osteogenic performance of the subchondral layer hydrogels in an inflammatory microenvironment. **A** ALP and ARS staining at 7 and 14 days under normal conditions (without H_2_O_2_) and staining at 14 days under inflammatory conditions (with H_2_O_2_); **B** qPCR results of Ocn, Opn, and Col I in BMSCs after treated with different hydrogels in an inflammatory environment (with H_2_O_2_); **C** Immunofluorescence staining of Ocn, Opn, and Col I in BMSCs after treated with different hydrogels in an inflammatory environment (with H_2_O_2_). The data are presented as the means ± standard deviations (SD), n = 3. (*p < 0.05; **p < 0.01; ****p < 0.0001)

In patients with OA or osteochondral injury, the subchondral bone is a very important and neglected component. Studies have shown that treating subchondral bone is also a potential therapeutic approach for treating OA. In our bilayer hydrogel, the subchondral layer not only contains nanozymes that play an antioxidant role but also contains hap nanoparticles, which can contribute to ossification. We first studied the osteogenic ability of the four groups of cells in a normal environment (without H_2_O_2_). ALP and ARS staining revealed that both the GA@H and GA@HLM groups exhibited strong osteogenic ability (Fig. 5A and Figure S13). However, in OA or OC patients, subchondral bone is often in an oxidative microenvironment, so it is important to study its osteogenic ability in this environment. After treatment with H_2_O_2_ (200 μM), the osteogenic benefits of GA@H disappeared, but the performance of GA@HLM was still superior (Fig. 5A and Figure S13). We then analysed the mRNA expression of three osteogenic genes, and the results showed that the expression of these genes was significantly upregulated only in the GA@HLM group (Fig. 5B). Similarly, the immunofluorescence results showed that the expression of Ocn, Opn, and Col I was significantly greater in the GA@HLM group than in the control group (Fig. 5C). Thus, in the oxidative stress microenvironment, the advantage of GA@HLM was demonstrated, which also indicated that GA@HLM has great potential for application in subchondral bone repair in patients with osteochondral injury.

**Fig. 6:**
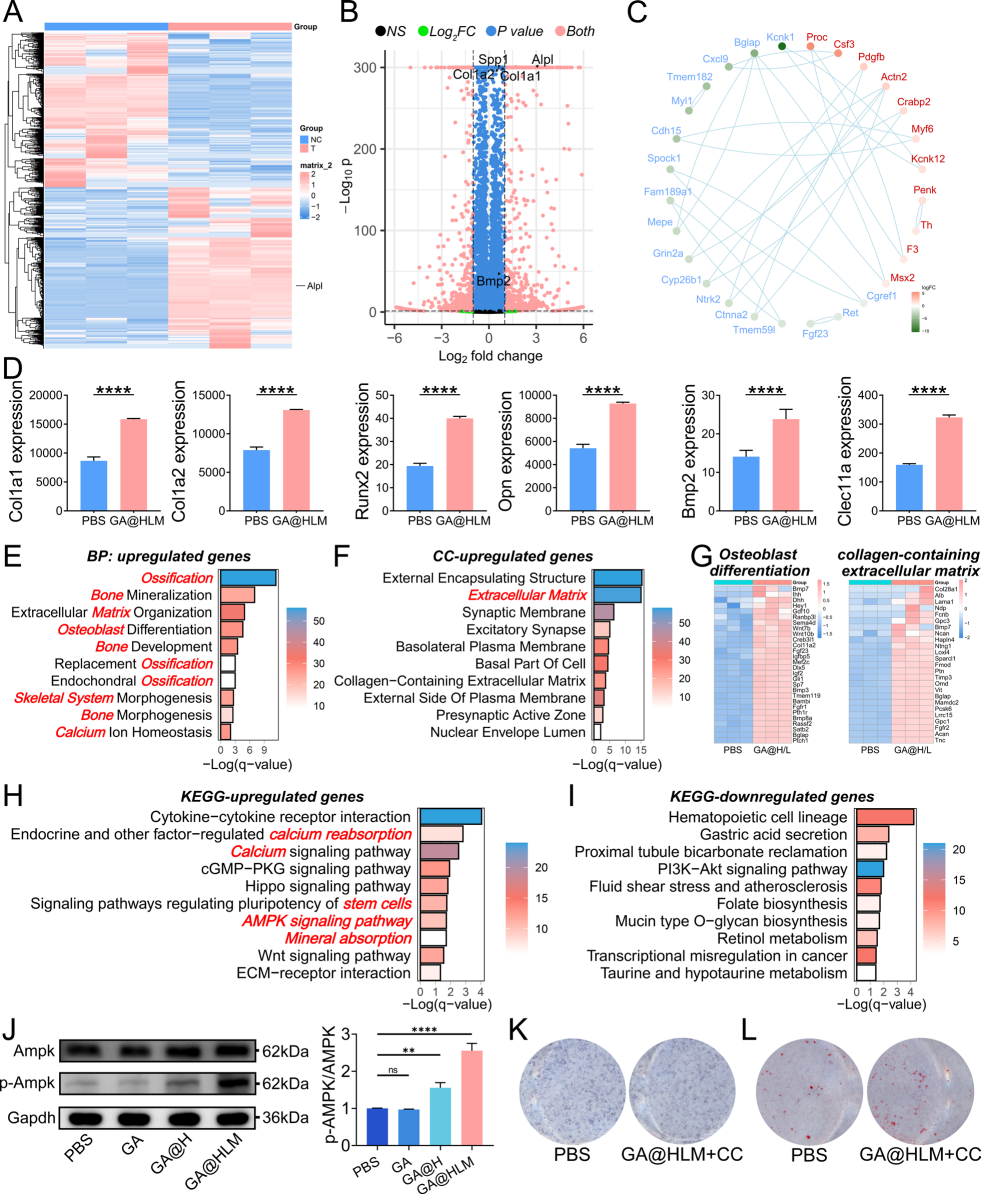
Analyses of the osteogenic mechanism of the GA@HLM hydrogel in an inflammatory microenvironment. **A** Heatmap showing DEGs between BMSCs treated with PBS and GA@HLM hydrogel; **B** Volcano plot showing the differential expression results; **C** Protein–protein interaction **D** Representative GO items (BP) of genes upregulated in the GA@HLM hydrogel-treated groups; **E** Representative GO items (CC) of genes upregulated in the GA@HLM hydrogel-treated groups; **F** RNA sequencing results of genes related to osteoblast differentiation and collagen containing the extracellular matrix; **G** Representative KEGG items of genes upregulated in the GA@HLM hydrogel-treated groups; **H** Representative KEGG items of genes downregulated in the GA@HLM hydrogel-treated groups; **I** RNA sequencing results for six genes related to bone metabolism; **J** The expression of AMPK pathway-related biomarkers; **K** and **L** ALP and ARS staining between the PBS-treated and GA@HLM hydrogel + Compound C-treated groups. The data are presented as the means ± standard deviations (SD), n = 3. (**p < 0.01; ****p < 0.0001)

To further elucidate the osteogenic mechanism of the GA@HLM hydrogel, we performed transcriptome sequencing. A total of 1540 genes were confirmed to be DEGs between the PBS and GA@HLM groups, including 753 genes upregulated in the GA@HLM group (Fig. 6A, B). The PPI results are shown in Fig. 6C. According to the differential expression analyses, although the LogFC values of some osteogenic genes did not meet the conditions for differential gene expression, their p values were significant (Fig. 6D). We speculate that this may be due to the small sample size (n = 3). Overall, the results of our transcriptome sequencing were exciting, and the expression of many osteogenic genes was elevated in the GA@HLM group. We then performed enrichment analyses, including GO and KEGG analyses. Interestingly, GO analyses revealed that the genes upregulated in the GA@HLM group were related to several osteoblast functions, such as ossification, bone mineralization, osteoblast differentiation, bone morphogenesis, and extracellular matrix-related functions (Fig. 6E, F). The gene expression patterns associated with osteoblast differentiation and collagen-containing extracellular matrix items are shown in Fig. 6G, and the results indicated that these genes were highly expressed in the GA@HLM group. According to the KEGG pathway analyses, several calcium ion-related pathways were enriched in the GA@HLM group (Fig. 6H). Interestingly, the AMPK signaling pathway, an osteoblast differentiation-related pathway, was significantly associated with the genes upregulated in the GA@HLM group [44, 45]. In contrast, among the genes downregulated in the GA@HLM group, we did not observe an association with osteogenic pathways (Fig. 6I). Furthermore, the role of the AMPK pathway in the osteogenic activity of GA@HLM was validated. The expression of AMPK pathway-related genes was significantly greater in the GA@HLM group than in the PBS group (Figure S14), and the activity of p-AMPK was significantly upregulated by GA@HLM (Fig. 6J). In addition, we added the AMPK inhibitor compound C to BMSCs [46–48]. Apparently, compound C effectively abolished the effect of the GP@HLM on osteogenesis (Fig. 6K, L). Taken together, the present findings suggested that GP@HLM treatment promoted osteoblast differentiation by activating the AMPK signaling pathway.

### In vivo studies confirmed that bilayer hydrogels are effective biomaterials for osteochondral regeneration

**Fig. 7:**
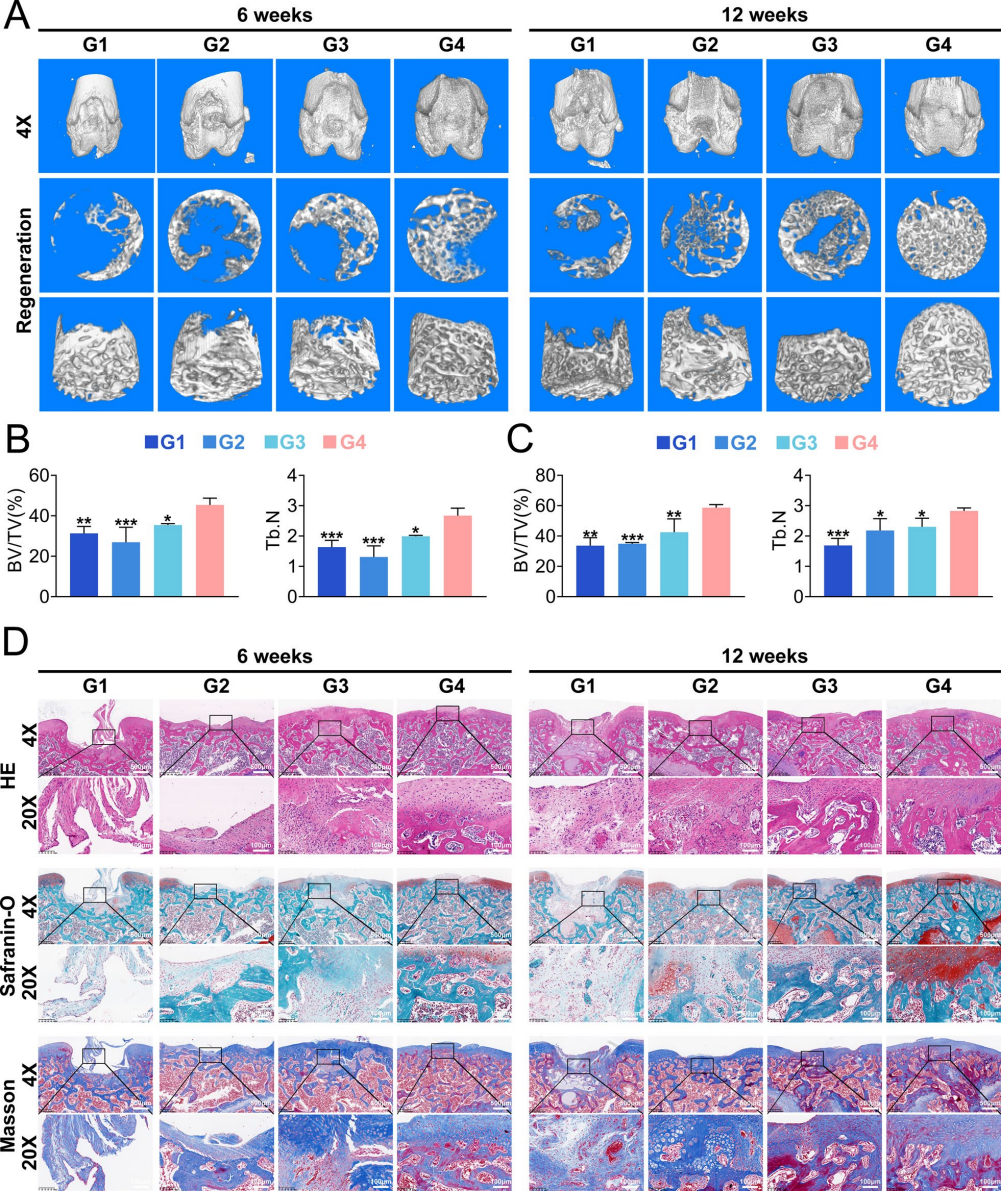
In vivo performance of the bilayer hydrogel scaffold in restoring osteochondral defects in rats. **A** Micro-CT images of rat osteochondral defects treated with different hydrogel scaffolds; **B** Quantitative analysis of micro-CT data for rat knee samples obtained at 6 weeks; **C** Quantitative analysis of micro-CT data for rat knee samples obtained at 12 weeks; **D** H-E, Safranin-O and Masson staining of rat knee osteochondral defects treated with different hydrogel scaffolds. G1: PBS; G2: GH + GA; G3: GH + GA@H; G4: GH@LM + GA@HLM. The data are presented as the means ± standard deviations (SD), n = 3. (*p < 0.05; **p < 0.01; ***p < 0.001)

The microCT results showed that the subchondral bone repair ability of the GH@LM + GA@HLM (G4) group was better than that of the other three groups, and the amount of new bone tissue in the GH@LM + GA@HLM (G4) group was greater than that in the other groups at 6 and 12 weeks (Fig. 7A). According to the quantitative results, both the BV/TV and Tb.N were significantly optimized in the GH@LM + GA@HLM (G4) group (Fig. 7B, C). Histological analyses, including HE, S–O and Masson staining, were used to evaluate the repair of osteochondral defects. H&E staining revealed almost no effective tissue repair in the control group, while the GH + GA (G2) and GH + GA@H (G3) groups exhibited partial tissue repair but still exhibited high fibrous tissue infiltration at 12 weeks. To test this hypothesis, the GH@LM + GA@HLM (G4) group exhibited more new tissue repair at the defect site (Fig. 7D). S–O green clearly showed cartilage and subchondral bone tissue, which is the most commonly used histological evaluation index for osteochondral repair. Although GH + GA@H (G3) promoted the repair of subchondral bone to a certain extent due to the presence of hap, the repair of the cartilage layer was still unsatisfactory. In contrast, the GH@LM + GA@HLM (G4) group exhibited complete subchondral bone and some cartilage repair at 6 weeks, and this group achieved integrated osteochondral repair at 12 weeks (Fig. 7D).

**Fig. 8:**
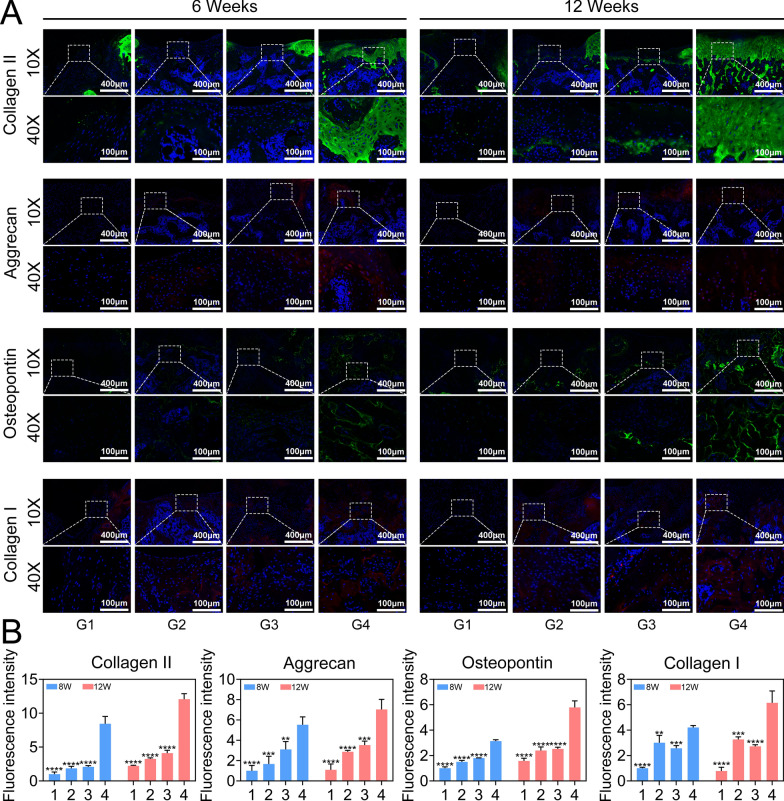
Immunofluorescence images (**A**) and statistical analyses (**B**) of rat knee osteochondral defects treated with different hydrogel scaffolds. G1: PBS; G2: GH + GA; G3: GH + GA@H; G4: GH@LM + GA@HLM. The data are presented as the means ± standard deviations (SD), n = 3. (**p < 0.01; ***p < 0.001; ****p < 0.0001)

We then performed immunofluorescence analysis to quantify the regeneration of subchondral bone and cartilage repair. Aggrecan and Col II were selected for use in evaluating cartilage, and Opn and Col I were selected for use in evaluating subchondral bone. Notably, the expression of Col II in the cartilage matrix was the highest in the GH@LM + GA@HLM group (G4), and Col II was still detected in only a small number of cells and surrounding cells at 12 weeks (Fig. 8A, B). Similarly, the number of aggrecan-positive cells was the highest in the GH@LM + GA@HLM group (G4). In subchondral bone, the levels of Col I and Opn, two osteogenic markers, were also significantly greater in the GH@LM + GA@HLM (G4) group than in the other three groups (Fig. 8A, B). Finally, in order to confirm the antioxidant capacity of our hydrogel in vivo, we performed immunofluorescence analysis of Sod2. The results showed that the expression of Sod2 was significantly higher in the GH@LM + GA@HLM (G4) group than in the other three groups (Figure S15). Generally, the results of in vivo studies indicated that the GH@LM + GA@HLM (G4) group has excellent osteochondral repair performance and is a potential drug for clinical application in osteochondral repair.

Finally, to examine the biotoxicity of the hydrogel in rat, hearts, livers, spleens, lungs, and kidneys were collected on the 12-week group. The H&E staining revealed no pathological changes in these organs, indicating that the composite hydrogel had favourable biological safety (Figure S16).

## Conclusion

In conclusion, this study developed a LiMn_2_O_4_ nanozyme-functionalized bilayer hydrogel scaffold to regulate the inflammatory microenvironment and the AMPK signaling pathway for rat osteochondral regeneration. The hydrogel scaffold has layered physical properties that mimic the anatomical osteochondral structure and can be used to effectively manipulate the special physiological environment of osteochondral defects. This scaffold can effectively eliminate ROS and promote cartilage regeneration and osteogenic differentiation of BMSCs at the subchondral bone. Notably, transcriptomics revealed the mechanism underlying the performance of our scaffold and strongly confirmed its reliability. Due to its excellent biocompatibility and adequate tissue repair capabilities, we believe this scaffold has great promise for future clinical treatment of osteochondral defects.

### Supplementary Information

Supplementary material 1.

## Data Availability

The data that support the findings of this study are available from the corresponding author upon reasonable request.

## Abbreviations

ROS: Reactive oxygen species
BMSC: Bone marrow mesenchymal stem cell
OA: Osteoarthritis
CAT: Catalase
POD: Peroxidase
SOD: Superoxide dismutase
IBD: Inflammatory bowel disease
Alg: Sodium alginate
Hap: Nanohydroxyapatite
FBS: Fetal bovine serum
TEM: Transmission electron microscopy
XRD: X-ray diffraction
XPS: X-ray photoelectron spectroscopy
SEM: Scanning electron microscopy
EDS: Energy dispersive spectrometer
RC: Rat chondrocyte
DEG: Differentially expressed gene
GO: Gene ontology
KEGG: Kyoto encyclopedia of genes and genomes
PPI: Protein–protein interaction
IACUC: Institutional Animal Care and Use Committee
BMD: Bone mineral density
BV: Bone volume
TV: Total volume
GSH: Glutathione
SD: Standard deviations
OSCAR: Osteoclast-related receptor
