# Correction: A nanozyme-functionalized bilayer hydrogel scaffold for modulating the inflammatory microenvironment to promote osteochondral regeneration

**DOI:** 10.1186/s12951-024-02768-y

**Published:** 2024-08-23

**Authors:** Chuan Hu, Ruipeng Huang, Jiechao Xia, Xianjing Hu, Dingqi Xie, Yang jin, Weiming Qi, Chengliang Zhao, Zhijun Hu

**Affiliations:** 1 Department of Orthopaedic Surgery, Key Laboratory of Musculoskeletal System Degeneration and Regeneration Translational Research of Zhejiang Province, Sir Run Run Shaw Hospital, Medical College of Zhejiang University, Hangzhou, 310016 China; 2 Department of Orthopaedic Surgery, the Affiliated Hospital of Qingdao University, Qingdao, 266000 China; 3 The Second Affiliated Hospital of Wenzhou Medical University, Wenzhu, 325000 China; 4 Zhejiang Center for Medical Device Evaluation, Zhejiang Medical Products Administration, Hangzhou, 310009 China

**Correction: Journal of Nanobiotechnology (2024) 22:445** 10.1186/s12951-024-02723-x

In this article, the supporting information published with only table S1 without 16 figures provided by the author. The supporting information has been corrected with 16 figures and one table (Table S1) in this correction and the original article has been corrected.

The publisher apologises to the authors and readers for the inconvenience caused by this error.

### Supplementary Information

Additional file 1.

