# Supplementary material for: A nanozyme-functionalized bilayer hydrogel scaffold for modulating the inflammatory microenvironment to promote osteochondral regeneration
Source: J Nanobiotechnology. 2024 Jul 28;22:445. doi: 10.1186/s12951-024-02723-x (PMC11283693; doi:10.1186/s12951-024-02723-x)
Supplement: Supplementary file 1 — Supplementary material 1. [file 12951_2024_2723_MOESM1_ESM.docx]

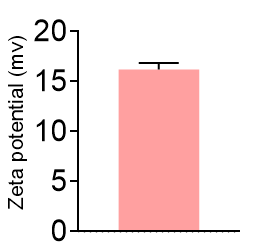


**Figure S1**. Zeta potential of LiMn_2_O_4._


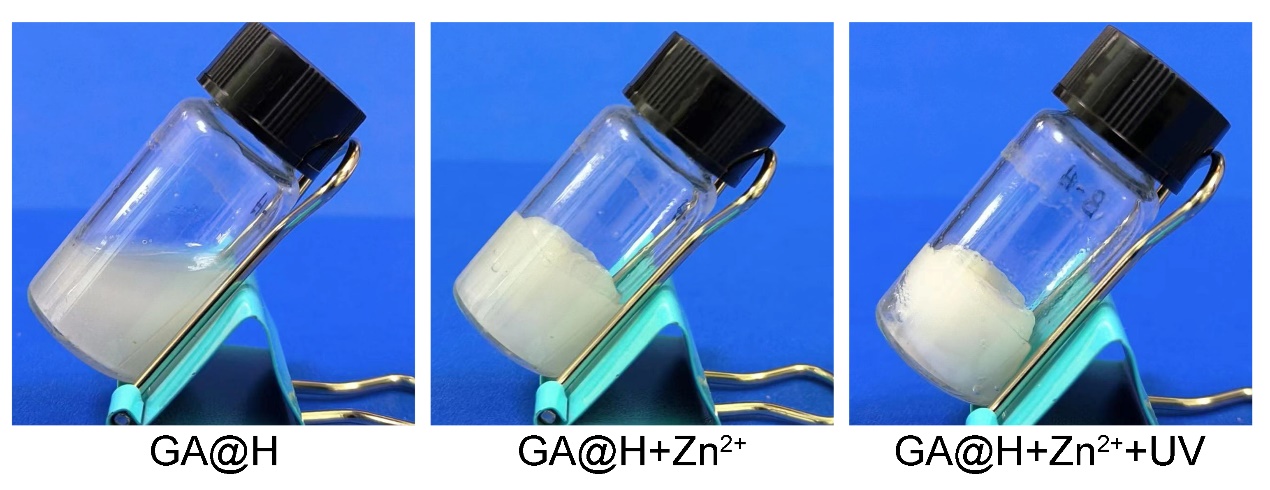


**Figure S2**. The photos of gel-sol transition about GA@H hydrogels.


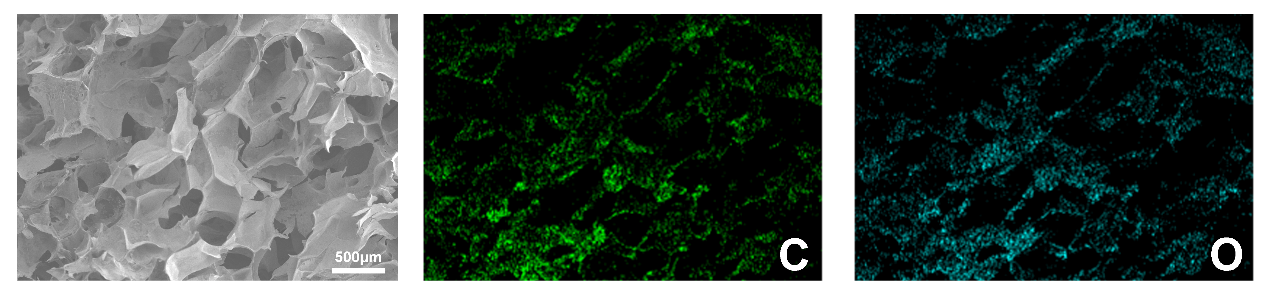


**Figure S3**. Elemental mappings of GH hydrogel.


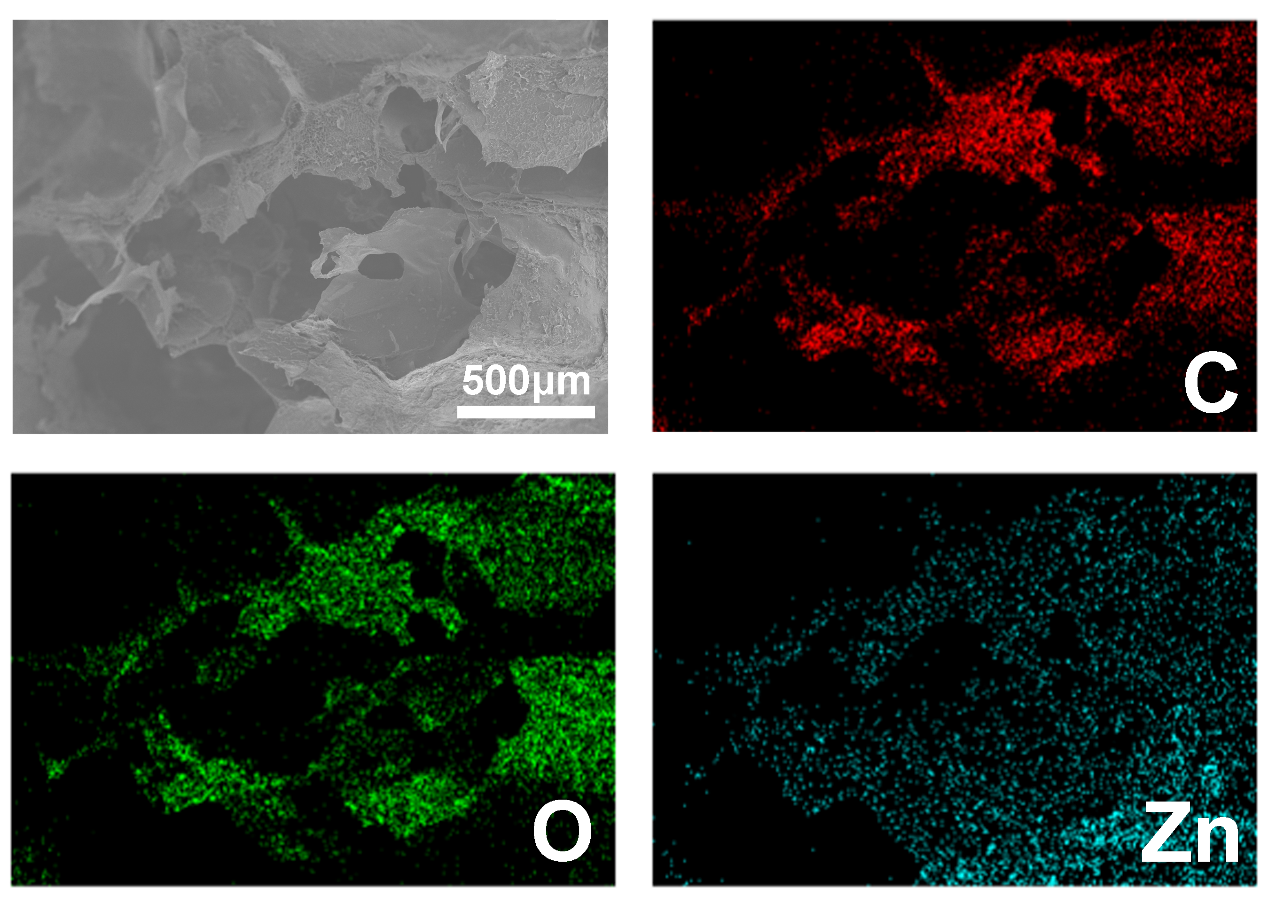


**Figure S4**. Elemental mappings of GA hydrogel.


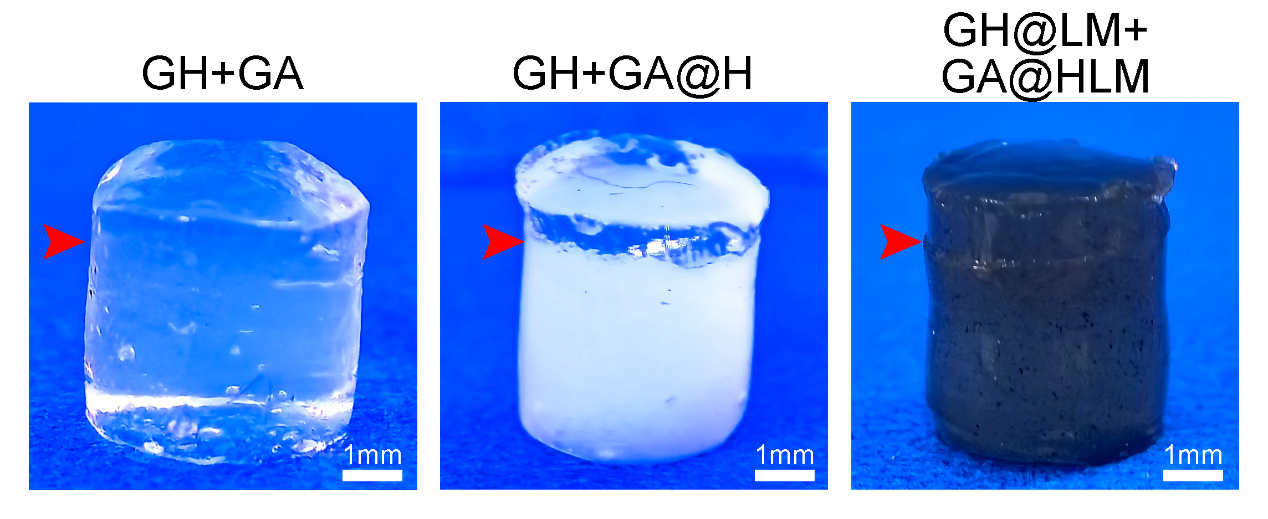


**Figure S5**. The photos of GH+GA, GH+GA@H, and GH@LM+GA@HLM hydrogels.


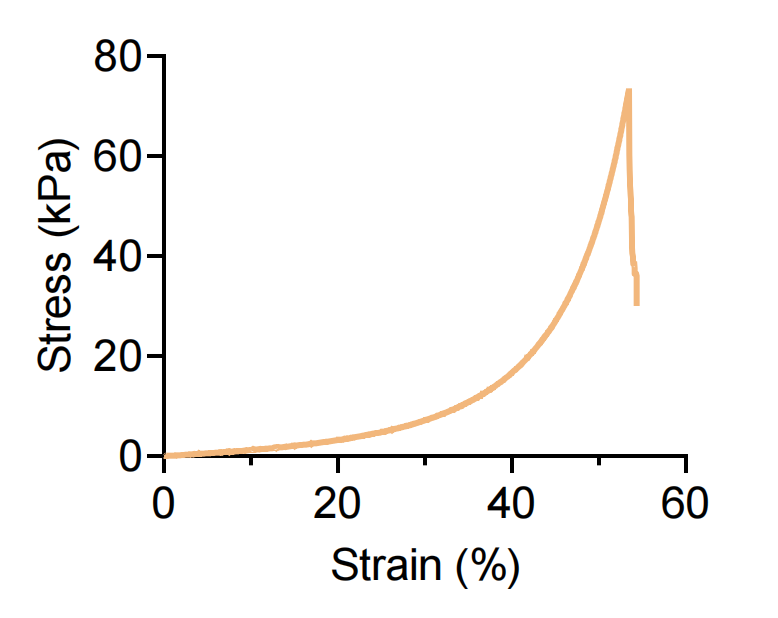


**Figure S6**. Mechanical testing of GH@LM+GA@HLM hydrogel.


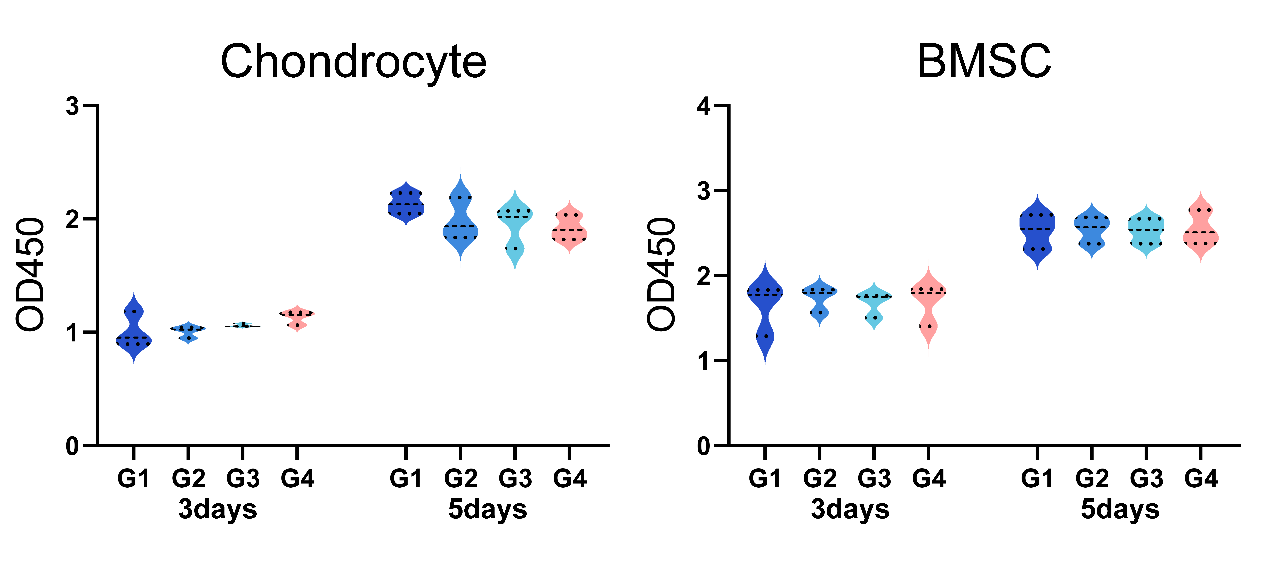


**Figure S7**. Cell Counting Kit-8 of cells treated with hydrogels. G1: PBS; G2: GH+GA; G3: GH+GA@H; G4: GH@LM+GA@HLM. Data are means ± standard deviation (SD), n=3.


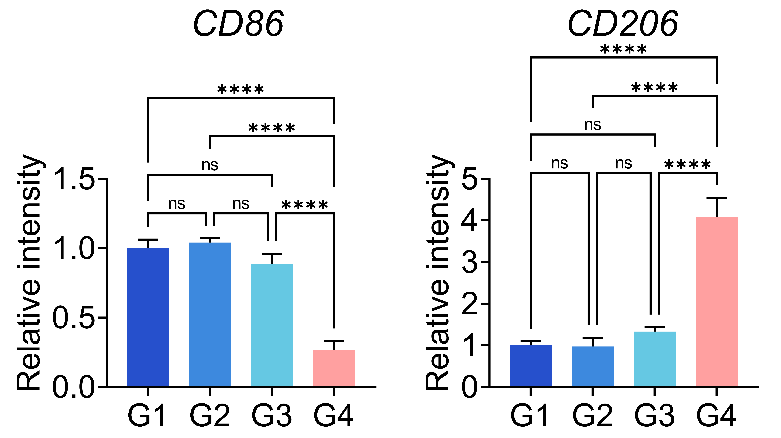


**Figure S8**. Quantitative analysis of polarization markers of macrophages treated with hydrogel scaffolds. G1: PBS; G2: GH+GA; G3: GH+GA@H; G4: GH@LM+GA@HLM. Data are means ± standard deviation (SD), n=3. (****, p < 0.0001).


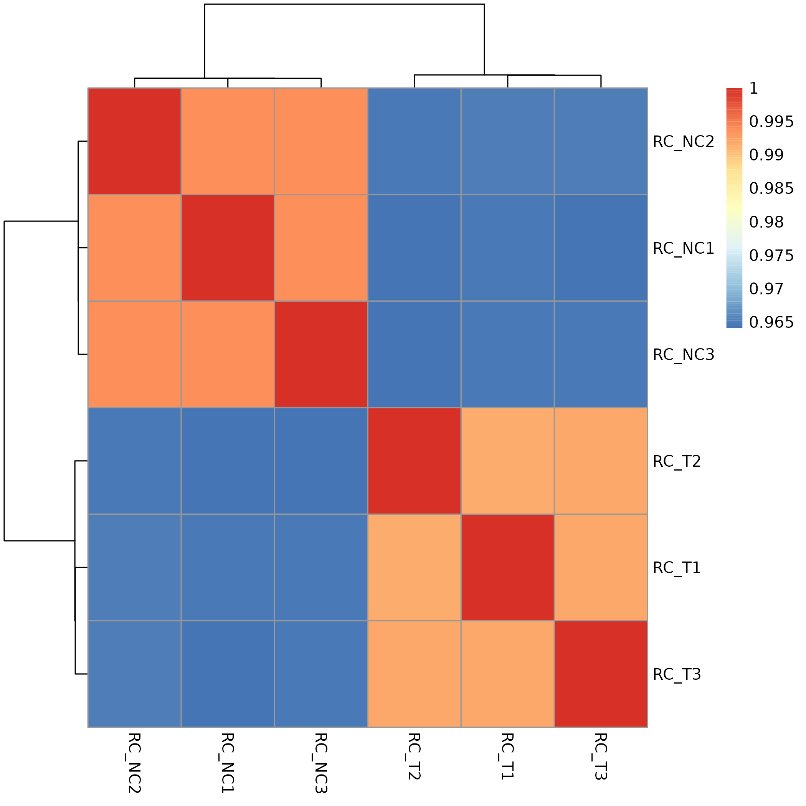


**Figure S9**. Correlation analysis of RNA sequencing samples.


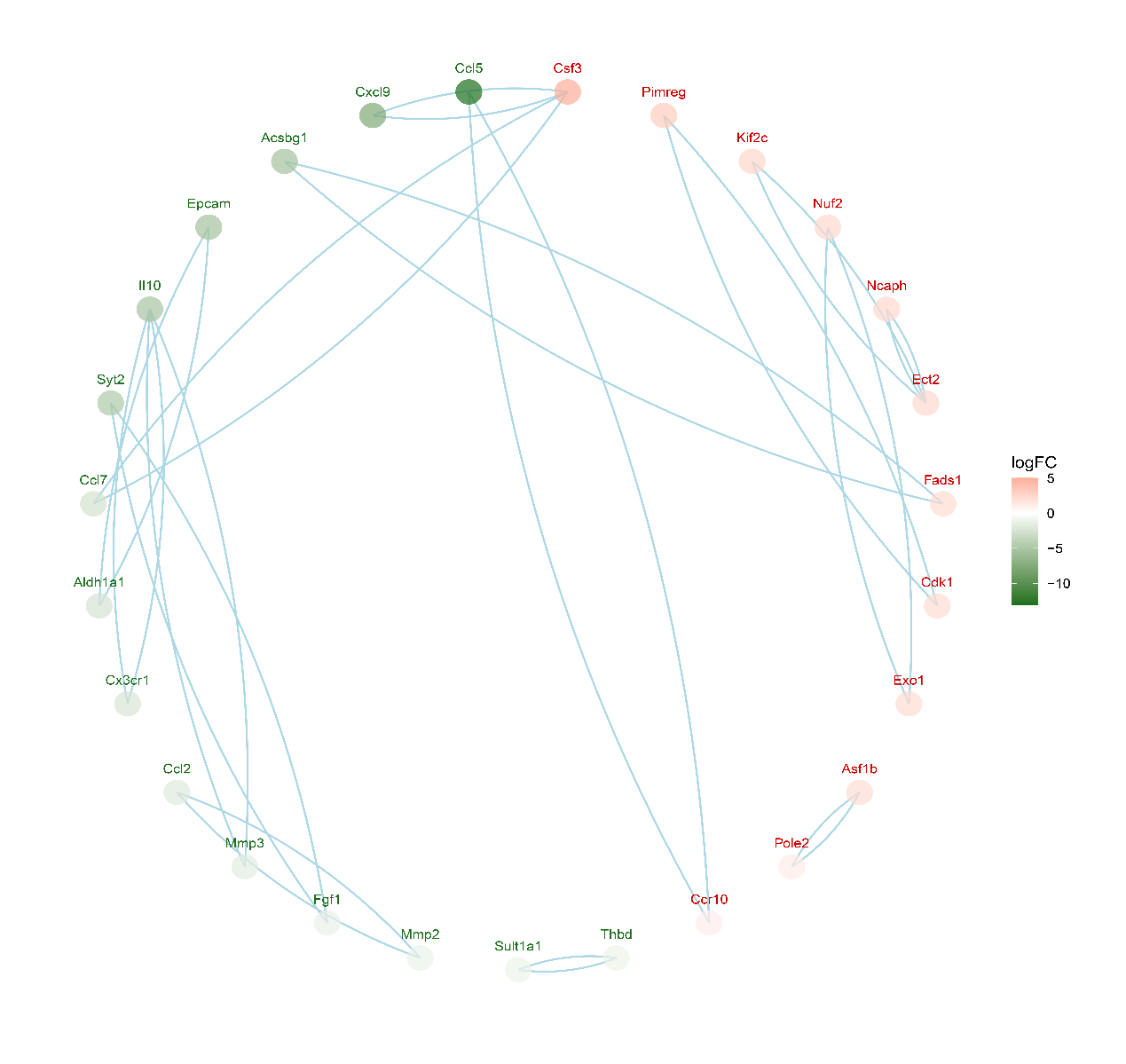


**Figure S10**. Protein protein interaction of differential expressed genes between chondrocyte treated with PBS or hydrogels.


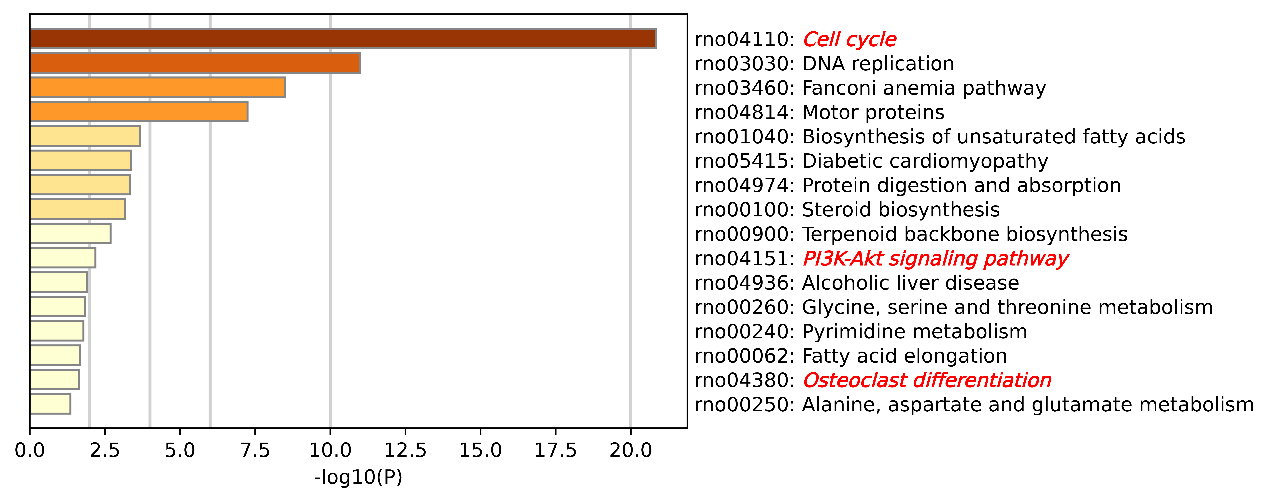


**Figure S11**. Representative KEGG items of genes down-regulated in GH@LM hydrogel treated chondrocytes.


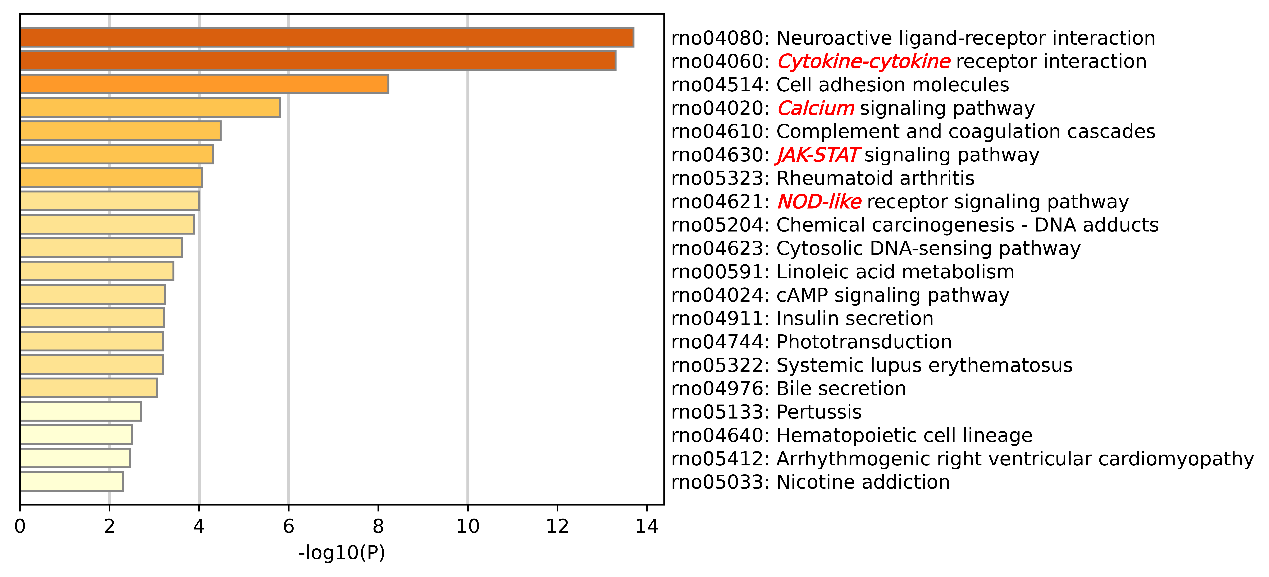


**Figure S12**. Representative KEGG items of genes up-regulated in GH@LM hydrogel treated chondrocytes.


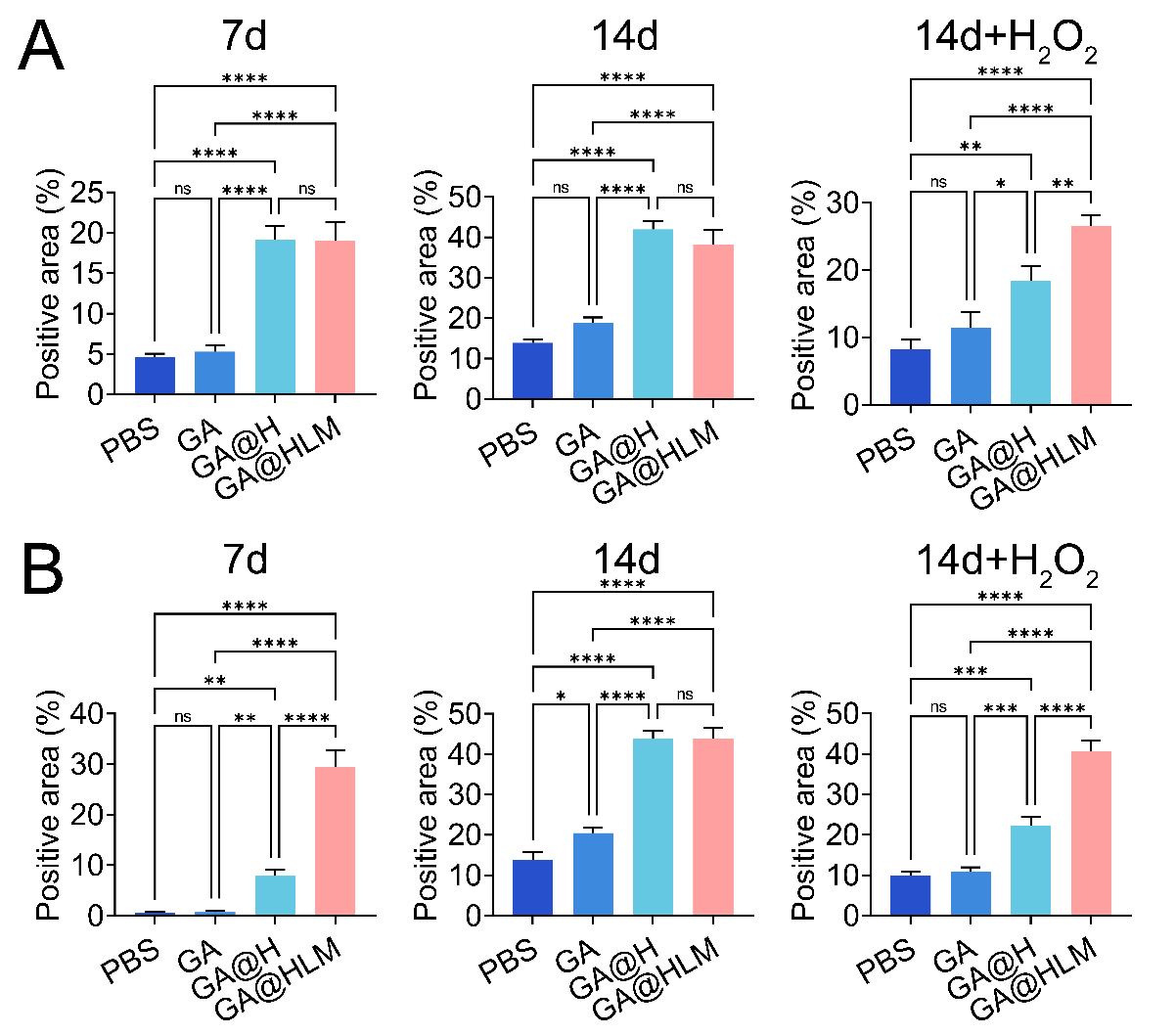


**Figure S13**. Statistical results of ALP (A) and ARS (B) staining. The data are presented as the means ± standard deviations (SD), n=3. (*, p < 0.05; **, p < 0.01; ***, p < 0.001; ****, p < 0.0001).


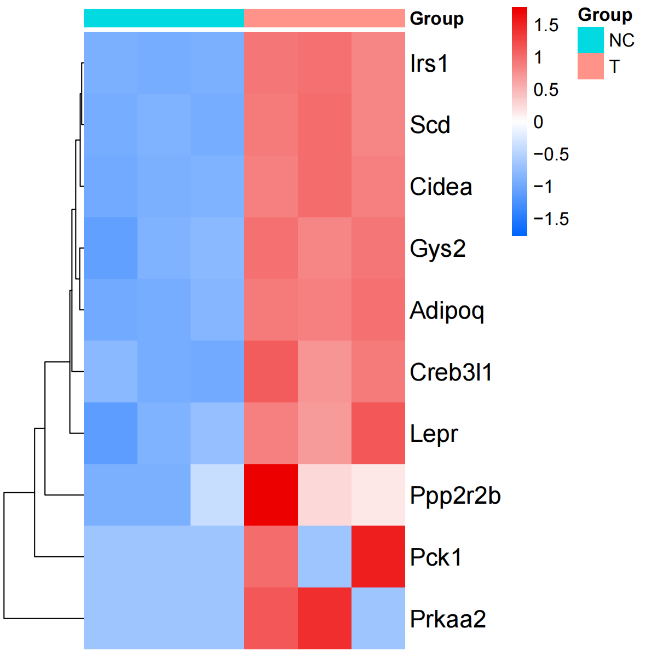


**Figure S14**. Heatmap showing the differences in the expression of AMPK pathway-related genes between BMSCs treated with PBS and those treated with the GA@HLM hydrogel.


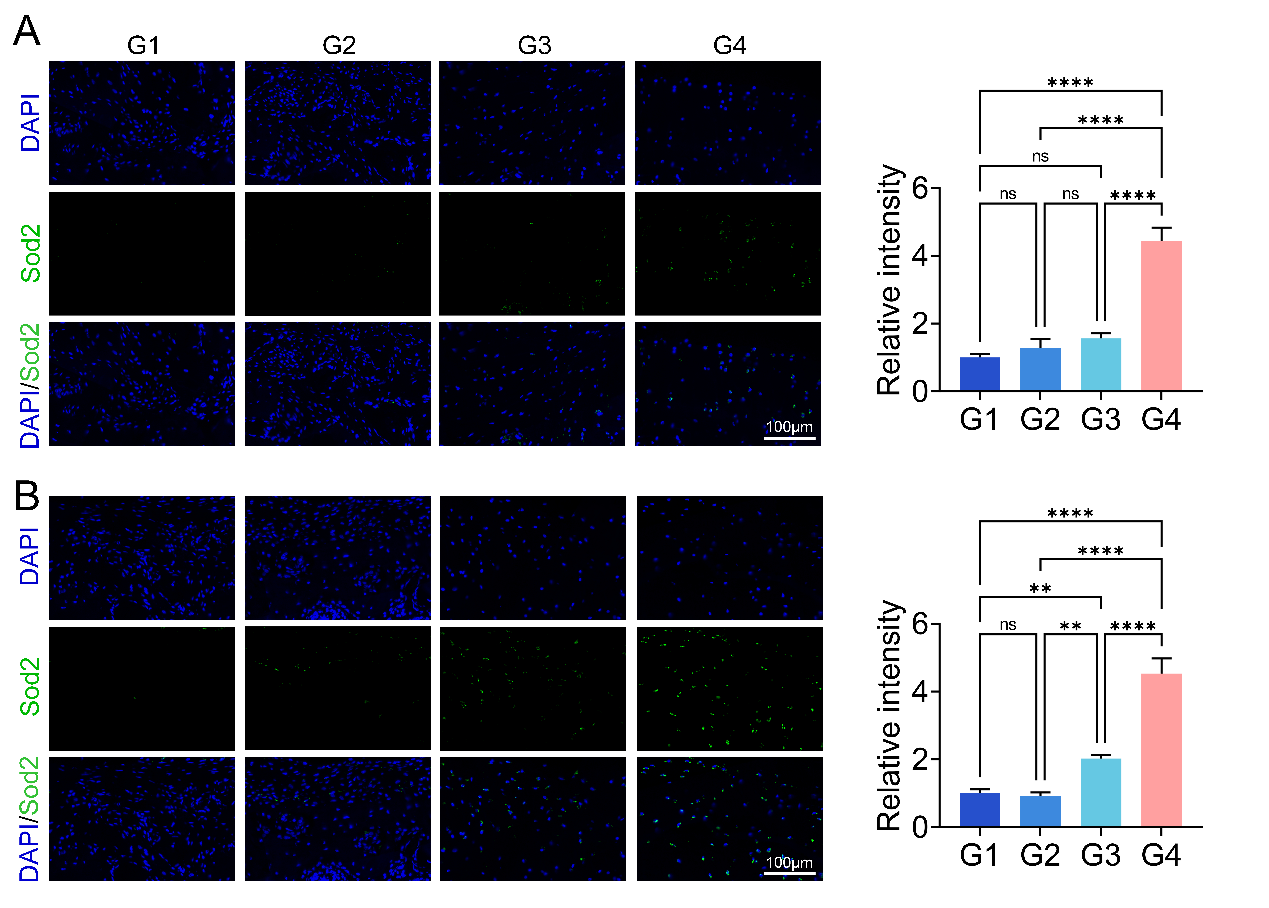


**Figure S15**. Immunofluorescence images of rat knee osteochondral defects treated with different hydrogel scaffolds. (A) 6 weeks; (B) 12 weeks G1: PBS; G2: GH+GA; G3: GH+GA@H; G4: GH@LM+GA@HLM. The data are presented as the means ± standard deviations (SD), n=3. (**, p < 0.01; ****, p < 0.0001).


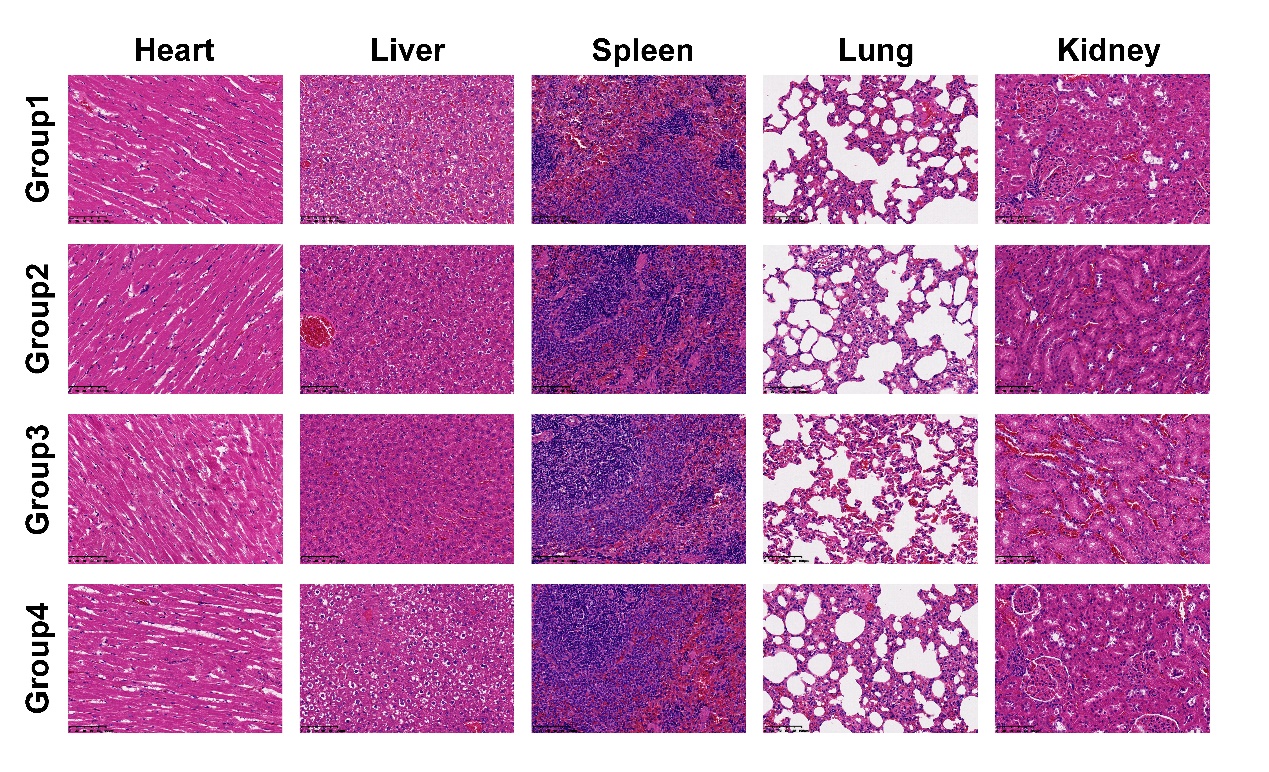


**Figure S16**. H&E staining of major organs to demonstrate the biosafety of hygrogels. Group1: PBS; Group2: GH+GA; Group3: GH+GA@H; Group4: GH@LM+GA@HLM.

**Table S1.** The primers used in the present study.

| R-Col2a1-F | CCTGGACCCCGTGGCAGAGA |
| --- | --- |
| R-Col2a1-R | GCAGGGCCAGAAGTACCCTGATC |
| R-Mmp13-F | CATCATCTGGGAGCATGAAA |
| R-Mmp13-R | GCAGCTCCAAAGGCTACAA |
| R-Aggr-F | TGGGGTCCGTGGGCTCACAA |
| R-Aggr-R | CATTCGCACGGGAGCAGCCA |
| R-Ocn-F | CAACCCCAATTGTGACGAGC |
| R-Ocn-R | GGCAACACATGCCCTAAACG |
| R-Opn-F | GGAGTCCGATGAGGCTATCAA |
| R-Opn-R | TCCGACTGCTCAGTGCTCTC |
| R-Col I-F | CAGGCTGGTGTGATGGGATT |
| R-Col I-R | CCAAGGTCTCCAGGAACACC |
| R-Sod2-F | GCTGGAGGCTATCAAGCGTGAC |
| R-Sod2-R | TTAGAGCAGGCGGCAATCTGTAAG |
| R-Cat-F | GGCCTGACTGACGCGATTGC |
| R-Cat-R | CTGCTCCTTCCACTGCTTCATCTG |
| R-Gapdh-F | TGGTGAAGGTCGGTGTGAAC |
| R-Gapdh-R | GGGATCTCGCTCCTGGAAGATG |
